# Insights into the Growth of Ternary WSSe Nanotubes in an Atmospheric CVD Reactor

**DOI:** 10.1021/acs.inorgchem.3c02903

**Published:** 2023-10-24

**Authors:** R. Rosentsveig, M. B. Sreedhara, S. S. Sinha, I. Kaplan-Ashiri, O. Brontvein, Y. Feldman, I. Pinkas, K. Zheng, I. E. Castelli, R. Tenne

**Affiliations:** † Department of Molecular Chemistry and Materials Science, Weizmann Institute of Science, Rehovot 7610001, Israel; ‡ Solid State and Structural Chemistry Unit, Indian Institute of Science, Bengaluru 560012, India; § Plasmon Nanotechnologies, Istituto Italiano Di Tecnologia, Via Morego 30, Genova 16163, Italy; ∥ Department of Chemical Research Support, Weizmann Institute of Science, Rehovot 7610001, Israel; ⊥ Department of Energy Conversion and Storage, Technical University of Denmark, DK-2800 Kgs Lyngby, Denmark

## Abstract

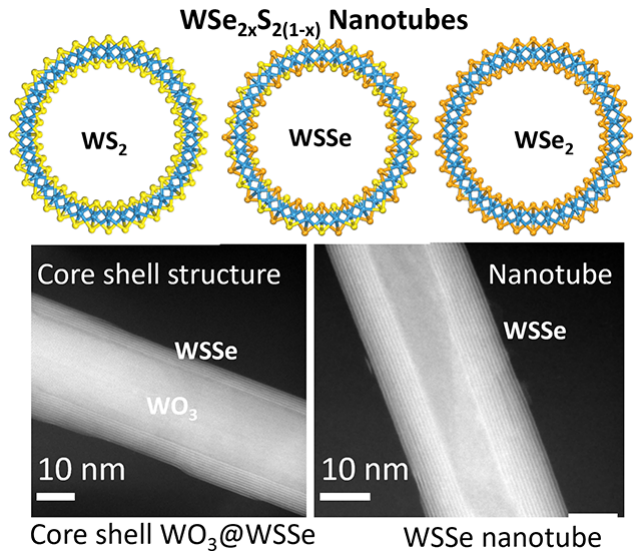

The synthesis of complex new nanostructures is challenging but also bears the potential for observing new physiochemical properties and offers unique applications in the long run. High-temperature synthesis of ternary WSe_2*x*_S_2(1–*x*)_ (denoted as WSSe) nanotubes in a pure phase and in substantial quantities is particularly challenging, requiring a unique reactor design and control over several parameters, simultaneously. Here, the growth of WSSe nanotubes with the composition 0 ≤ *x* < 1 from W_18_O_49_ nanowhiskers in an atmospheric chemical vapor deposition (CVD) flow reactor is investigated. The oxide precursor powder is found to be heavily agglomerated, with long nanowhiskers decorating the outer surface of the agglomerates and their core being enriched with oxide microcrystallites. The reaction kinetics with respect to the chalcogen vapors varies substantially between the two kinds of oxide morphologies. Insights into the chemical reactivity and diffusion kinetics of S and Se within W_18_O_49_ nanowhishkers and the micro-oxide crystallites were gained through detailed microscopic, spectroscopic analysis of the reaction products and also through density functional theory (DFT) calculations. For safety reasons, the reaction duration was limited to half an hour each. Under these circumstances, the reaction was completed for some 50% of the nanotubes and the other half remained with thick oxide core producing new WO_*x*_@WSSe core–shell nanotubes. Furthermore, the selenium reacted rather slowly with the WO_*x*_ nanowhiskers, whereas the more ionic and smaller sulfur atoms were shown to diffuse and react faster. The yield of the combined hollow and core–shell nanotubes on the periphery of the agglomerated oxide was very high, approaching 100% in parts of the reactor boat. The nanotubes were found to be very thin (∼80% with a diameter <40 nm). The optical properties of the nanotubes were studied, and almost linear bandgap modulation was observed with respect to the selenium content in the nanotubes. This investigation paves the way for further scaling up the synthesis and for a detailed study of the different properties of WSSe nanotubes.

## Abstract 2

WSe_2*x*_S_2(1−*x*)_ (denoted as WSSe) nanotubes with 0 ≤ *x* < 1 were prepared at high temperatures by reacting W_18_O_49_ nanowhiskers with H_2_S gas and Se vapors in a reducing atmosphere. Partially reacted core−shell WO_3−*x*_@WSSe nanotubes were prepared as well. Some of the optical properties of the nanotubes were studied as well. Thorough analyses of the nanotubes were undertaken, and the growth mechanism is discussed in detail.

## 1 Introduction

Following the discovery of carbon nanotubes in 1991,^1^ nanotubes from the layered inorganic compounds WS_2_,^2^ MoS_2_^3^, and BN^4,5^ were also unveiled. The dominant mechanism for folding and seaming the layers is healing of the dangling bonds on the rim of the nanoplatelets. It was shown that the elastic energy of folding of the layers is more than compensated by seaming and healing the dangling bonds of the rim atoms with a net energy gain. Ab initio calculations showed that the overall total energy of the nanotubes is smaller than that of the respective nanoribbon over a range of diameters, but is always larger than the macroscopic platelet for any diameter.^6,7^ This implies that the nanotube is stable relative to the nanoribbon of the same size at small diameters, but is unstable compared to an infinite layer of the same composition.^6,7^ Furthermore, the folding energy of WS_2_ and MoS_2_ plane is about 10 times larger than that of the graphitic layer of carbon nanotubes.^7^ To comply with the large elastic energy of folding, WS_2_ (MoS_2_) nanotubes adopt a larger radii than their carbon counterparts and generally come in multiwall structure.^6^ This is, however, not the case with single-wall asymmetric (Janus) nanotubes, which exhibit a clear minimum in the total energy–size relationship for structures, i.e., they present a global minimum of this compound at a given size.^8−10^

The synthesis of multiwall WS_2_ nanotubes received a lot of attention in recent years,^11−13^ due to largely their interesting optical, electrical, and electromechancial properties. Several chemical strategies were conceived to facilitate folding of the layers and form pure phases of nanotubes from inorganic layered compounds. Arguably, the most successful reaction is the sulfurization of W_18_O_49_ (Mo_4_O_11_) nanowhiskers, which can be considered as a sacrificial nanotemplate for the synthesis of multiwall WS_2_,^14,15^ MoS_2_^16^, and WSe_2_^17,18^ nanotubes. This strategy led to the scaling-up of the production of pure WS_2_ nanotubes to semi-industrial quantities,^19^ permitting their extensive characterization and study of their physicochemical properties.

Recent studies revealed that WS_2_ nanotubes exhibit unique quasi-one-dimensional (quasi-1D) properties. The loss of inversion and time-reversal symmetry in such multiwall and chiral nanotubes induce quantum-mechanical properties, which are not typical for single-wall tubes and two-dimensional (2D) flakes. Thus, liquid ion gating of such nanotubes at cryogenic temperatures led to diameter-dependent superconductivity.^20,21^ The Little Parks oscillations prompted by an axial magnetic field are a hallmark of their 1D superconducting character. These nanotubes also displayed a strong bulk photovoltaic effect,^22^ nonetheless exhibiting efficiencies largely inferior to the Schockley–Queisser solar-to-electrical efficiency limit and commercial photovoltaic cells. Gallery whispering modes produced by light excitation of MoS_2_ nanotubes induced strong light–matter interaction with the excitons, forming polaritonic quasi-particles with apparent overtones in the nanotube’s photoluminescence.^23^ Similarly, a strong coupling effect and enhanced light scattering were revealed in the extinction of irradiated WS_2_ nanotubes with diameters >80 nm. Contrarily, nanotubes with a diameter smaller than 60 nm could not confine the light cavity modes and displayed only pure absorption.^24,25^ Single-level quantum transport was observed in MoS_2_ nanotubes with Bi contacts at temperatures below 100 mK.^26^ Light-induced ferroelectric effect was recently demonstrated in electrically biased WS_2_ nanotubes, and a memory device was fabricated by establishing a matrix of 4 × 4 pixels from an assortment of such nanotubes.^27^ The light-induced ferroelectric effect emerged from an interlayer contraction–expansion and a stick–slip mechanism between the different nanotube walls upon applying opposing biases. WS_2_ nanotube-based torsion resonators with potential applications as nanosensors and actuators were also recently reported.^28^ These few examples show the relevance of multiwall metal dichalcogenide nanotubes in electronics, mechatronics, and optoelectronics applications. Since these enticing electronic and optical properties are guided by the band structure (in addition to geometrical effect), tuning the band position of the multiwall nanotubes by compositional variation holds great academic and applied interest.

The study of nanotubes from ternary metal dichalcogenide compounds is of particular interest for tuning the band structure and properties. Most enticing among them are asymmetric (Janus) Se–W–S nanotubes, where sulfur occupies concave sites and selenium occupies a convex position of the tube wall. This geometry would bring entirely new kinds of materials with fascinating properties. Nanotubes from Janus layered structure, like Se–Mo–S, have been discussed by theorists in the literature quite extensively recently.^9,29,30^ Here, the asymmetry between the outer selenium layer and the inner sulfur layer would elicit folding of the layers and seaming the dangling bonds at the edges, much like halloysite,^31,32^ immogolite^33^ and misfit compounds^34^ do. Practically though, so far random distribution of the sulfur and selenium in the lattice was experimentally observed in ternary WSSe nanotubes.^35,36^ However, recently misfit nanotubes of the kind LaS-TaSe_2_ and LaS-(TaSe_2_)_2_ with highly asymmetric structure and large (local) dipole moment were produced by careful control of the S/Se ratio as well as the other reaction parameters.^37^ Notwithstanding the high temperature of the reaction (825–1100 °C), the large reaction enthalpy drove it to a highly selective and specific path.

In another study, Mo_0.56_W_0.44_S_2_ nanotubes were synthesized by the chemical vapor transport technique.^38^ The work function of these nanotubes is inferior to that of the pure binary nanotubes. Consequently, they showed excellent field emission properties. Recently, WSe_2*x*_S_2(1–*x*)_ (0 ≤ *x* ≤ 1) nanotubes were synthesized in a closed-ampule reaction between W_18_O_49_ nanowhiskers and sulfur–selenium mixture in different proportions.^36^ The bandgap of the nanotubes was tuned from ca. 1.98 to 1.54 eV by varying the sulfur-to-selenium ratio.

Preliminary examination of the ternary WSSe nanotubes grown in a chemical vapor deposition (CVD) reactor showed that the chemical reactivity of sulfur and selenium varied largely toward the WO_*x*_ whiskers, affording the control of the stoichiometry rather difficult. In addition, due to the sluggish kinetics and limited reaction time, the products are composed of both core–shell structures (a few WS_2_/WSe_2_ layers decorating the WO_*x*_ whiskers) and hollow nanotubes. Nonetheless, the present study portrays a route to obtain ternary WSe_2*x*_S_2(1–*x*)_ nanotubes in a flow reactor in the composition range covering 0 ≤ *x* < 1 via sulfurization/selenization using W_18_O_49_ nanowhiskers. This methodology is prone to many snags but is nonetheless highly valuable in the effort to scale up the production of such nanotubes. Various flux rates of sulfur and selenium were employed to optimize the composition and gain insight into the growth, structure, and optical properties of the ternary nanotubes. Acumen insight into the growth mechanism was obtained using advanced microscopic and spectroscopic tools. The nature of bonding and the difference in reactivity between sulfur and selenium with respect to WO_*x*_ were calculated using quantum-mechanical simulation. Surprisingly, unlike the pure WS_2_ nanotubes, the core–shell structures reported here did not display a strong optical coupling, which was attributed to the small average diameter of the nanotubes and the low refractive index of the tungsten oxide core.

## 2 Experimental Section

### 2.1 Synthesis-General

Tungsten oxide nanowhiskers, i.e., W_18_O_49_ (WO_2.72_), were used as a precursor for the sulfurization-selenization process. Such nanowhiskers were used in the past for different synthetic processes of WS_2_^14,15,19^ and WSe_2_^17,18^ nanotubes. Therefore, the synthesis of the oxide nanowhiskers will be briefly described, first. The characterization tools used in this work are described in detail in the Supporting Information (SI).

### 2.2 Synthesis of WO_2.72_ Nanowhiskers

Fast growth of the suboxide (W_18_O_49_ = WO_2.72_) nanowhiskers was accomplished through the following steps: reduction, sublimation, and condensation.^14,19^ For this synthesis, a precursor of WO_2.92_ nanoparticles was used. Figure S1a (see the SI) displays the scanning electron microscopy (SEM) image of agglomerated WO_2.92_ powder at low magnification, while the magnified Figure S1b shows the SEM of WO_2.72_ on the surface of the WO_2.92_ powder. The heavily agglomerated oxide powder has an important bearing on the reactions described below. The synthesis was carried out in a horizontal CVD furnace using a quartz tube reactor under a slightly reducing atmosphere.

For the nanowhiskers synthesis, the precursor powder, i.e., WO_2.92_ powder, was placed into a quartz boat. The reactor was purged continuously with nitrogen gas. The total gas flow rate was about 100–150 cm^3^/min^–1^. The flow rate of the hydrogen varied slightly between 0.5 and 5 cm^3^ min^–1^, with the rest being the nitrogen carrier gas. The boat was pushed to the hot zone of a horizontal furnace, which was preheated to 780–840 °C. The reactants were maintained at this temperature for 15–30 min. Once the reaction terminated, the boat was withdrawn from the furnace and left to cool down naturally to room temperature under the flow of nitrogen gas. The best product for the next step was obtained under the following parameters: about 0.5% H_2_ in the reaction gas mixture and a relatively low temperature (780 °C) for 15 min.

The whiskers were characterized by X-ray diffraction (XRD) and SEM and were subsequently used for the synthesis of WSe_2*x*_S_2(1–*x*)_ (0 ≤ *x* ≤ 1) nanotubes. Figure S2 shows SEM images of tungsten oxide powder at the end of the reaction. The overall shape of the agglomerated powder in low magnification looked quite the same as the original WO_2.92_ powder (Figure S2a). Presumably, the inner content of the agglomerate contains micrometer-sized oxide particles, which react quite differently with the chalcogen vapors at elevated temperatures. The contour of the oxide agglomerate is decorated with a dense web of W_18_O_49_ nanowhiskers (see Figure S2b). The whiskers were 25–100 nm in diameter and half to a few micrometers long. Figure S2c presents the XRD pattern of oxide nanowhiskers (space group *P*2/*m a* = 1.83, *b* = 0.38, and *c* = 1.4 nm), which can be assigned to the W_18_O_49_ phase.^39^ The size difference between the W_18_O_49_ crystallites in the center of the agglomerate and the surface-rich nanowhiskers has paramount importance on the reaction kinetics of the follow-up reaction with sulfur and selenium vapors and, consequently, on the product composition.

### 2.3 Synthesis of WSe_2*x*_S_2(1–*x*)_ (0 ≤ *x* ≤ 1) Nanotubes in the Flow Reactor

**Figure 1:**
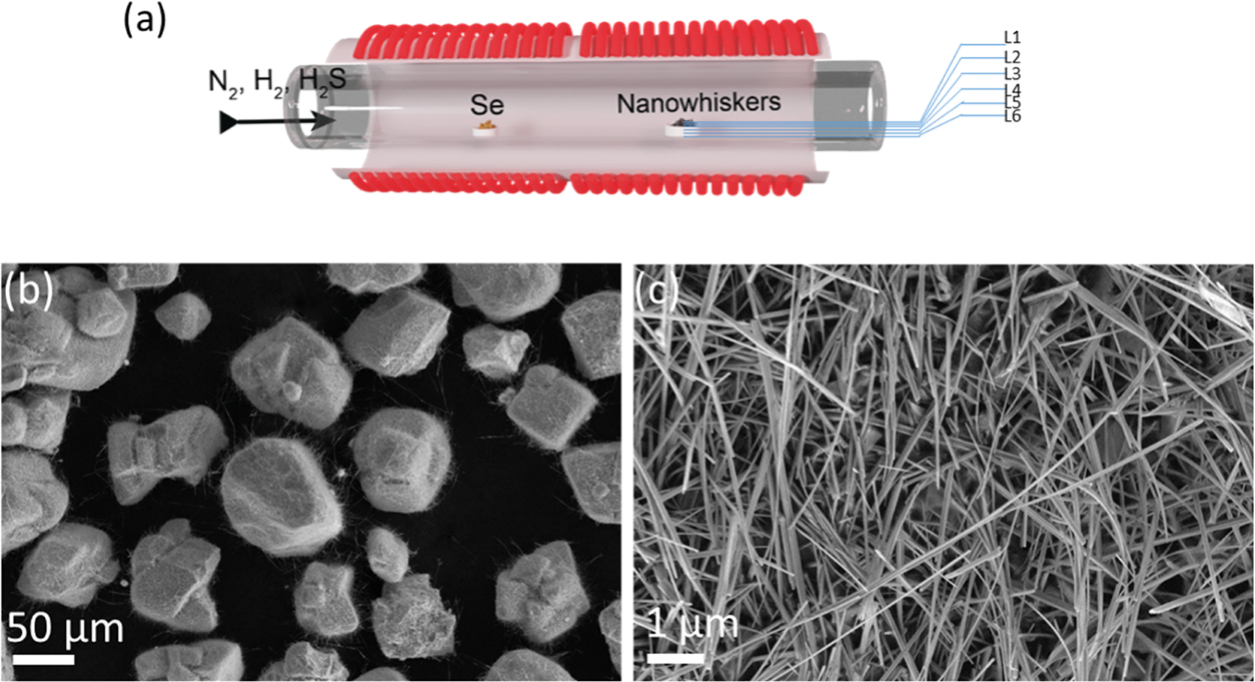
(a) Schematics of the CVD flow reactor used for the sulfurization/selenization process. The crucibles with selenium and WO_2.72_ whiskers are shown (for details, see Figure S3). The layers, which were peeled off from the product after the completion of the reaction, are indicated as L1 to L6. L1 is the top layer, and L6 is the deepest. (b, c) Low- and high-magnification SEM image of ternary WSSe nanotube agglomerates from batch E-2 (i.e., layer 2 of sample E). The agglomerates are very porous and constitute mostly nanotubes.

Figure 1a shows the scheme of the flow reactor used for the synthesis of WSSe nanotubes. The W_18_O_49_ nanowhiskers and selenium precursor powders were placed in separate quartz crucibles. The crucibles were placed in a quartz boat outside the hot zone of the reactor. The distance between the two crucibles was about 20–22 cm. The crucible containing the oxide precursor was ∼3.5 mm deep and contained ∼0.3 g of WO_2.72_ powder. The reactor was purged with forming gas and H_2_S prior to the reaction. Then, the boat with crucibles filled with the precursor powders was pushed into the heated zone. The selenium powder was located downstream of the oxide and was heated to 330–450 °C (Figure S3). The temperature profile was constant at the center of the furnace, where the oxide precursor was placed, and was varied in the range of 780–840 °C. The overall reaction can be summarized as shown in eq 1
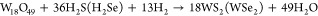
1The total gas flow rate was about 90–160 cm^3^ min^–1^. The flow rate of the hydrogen varied between 2 and 8 cm^3^ min^–1^ and that of H_2_S between 2 and 10 cm^3^ min^–1^, with the rest being nitrogen. No uncommon hazards are noted in this reaction. To determine the evaporation rate of selenium, the amount of selenium and the crucible were weighed first. Subsequently, the crucible was weighted after the reaction. The evaporation rate of the selenium varied between 2 and 21 mg min^–1^. The reaction took place for 30 min, and then, the boat was retracted back, moving the crucibles out of the heated zone. The reaction product was left to cool to room temperature naturally. Note that the kinetics of the reaction depends strongly on the thickness of the nanowhiskers, being rather slow for diameters larger than 60 nm. In order to limit the consumption of the toxic selenium (and H_2_Se vapors), the reaction was stopped after half an hour. Therefore, the oxide core remained in the center of about 50% of the tubes. In principle, it would be possible to complete the reaction here and get the hollow nanotubes by consuming large amounts of toxic selenium or continuing it in a closed ampule once the first few WSe_2*x*_S_2(1–*x*)_ on top of the oxide nanowhisker core have been obtained. The different batches of nanotubes are named A (almost pure tungsten sulfide) to F (enriched with respect to selenium), according to the selenium content in the phase (Table 1). The selenium fraction in the phase is defined as *x*_Se_ = Se/(Se + S), where Se and S stand for the concentrations in at% of the selenium and sulfur in the product.

**Table 1:** Summary of the SEM-EDS Composition Studied for the Various Samples at the Core of the Agglomerates and on the Periphery of the Agglomerates, Where Nanotubes Were Abundant^a^

sample	*x*_Se_: periphery of agglomerate	*x*_Se_: center of agglomerate	O/W: periphery of agglomerate	O/W: center of agglomerate	O/(S + Se): periphery of agglomerate	O/(S + Se): center of agglomerate
A-2	0.03	0.04	0.70	0.49	0.40	0.36
B-2	0.09	0.11	1.78	0.61	1.87	0.6
C-2	0.34	0.33	1.29	0.28	1.29	0.17
D-2	0.48	0.43	1.01	0.51	1.04	0.49
E-2	0.53	0.51	1.64	0.68	1.35	0.22
F-2	0.88	0.87	1.61	0.73	2.27	0.79

a The EDS data represents the ensemble average of the analysis on several agglomerates. Refer to SI Table S2 for a more detailed elemental analysis.

For the analytical work discussed below, the reaction product in the quartz boat was carefully peeled off by a spatula layer by layer from the top surface, and each layer was about 0.5–0.7 mm thick (see Figure 1a, layers L1 to L6). The first layer close to the surface (L1) was about twice thicker than the layers beneath, and the layers beneath are enumerated L2 to L6. While most of the analyses were focused on layers L2–L4, the diameter statistics (Table S1) were made on layers L2–L6. Accordingly, the batches are marked as A-1 to F-4 in Table 1. A–F represent the different compositions, with the richest in sulfur being A and the richest in selenium F. For example, C-2 is the second layer peeled from phase C and contains 33% selenium and 77% sulfur (Table 1). 1–6 is the layer number pealed from the respective sample with the top one- L1 and the deepest one – L6. In fact, the first layer at the top surface of the crucible (L1) serves as an effective diffusion barrier, somewhat analogous to the porous quartz filter used in the reactor described in ref (16). Therefore, in this top layer, the sulfurization/selenization reactions proceeded very fast, and hardly any nanotubes were obtained there. WSSe nanotubes were abundant in the powder beneath the top layer (layers 2–6). In the fourth layer and lower, the sulfur and especially the selenium were scarce, and much oxide was left after the 30 min reaction. The spectroscopic and microscopic tools used to characterize the structure, composition, and properties of the nanotubes are described in the SI.

### 2.4 Quantum-Mechanical Simulation of the WSSe Formation

The formation mechanism of WSSe nanotubes was investigated via materials modeling in the framework of density functional theory (DFT) using the Vienna Ab initio simulation package (VASP) package.^40^ Geometric relaxations and electronic band structures were calculated using the Perdew–Burke–Ernzerhof (PBE)^41^ functional in the projector augmented wave (PAW) mode with a cutoff energy of 500 eV, which was applied to deal with the interaction between ions and valence electrons. The thickness of the vacuum layer was set to 20 Å to avoid interaction between two adjacent layers. The formation mechanism is investigated on WO_3_(001) as the substitute of WO_2.92_ to make the model more feasible although realistic. During structural optimization, energy and force convergence criteria were set to 10^–6^ and 10^–2^ eV/Å. The Brillouin zone was sampled by the Monkhorst–Pack γ-centric k-point mesh with a density of 2π × 0.02 Å^–1^. The LOBSTER package^42^ was used to analyze the strength of the bonds for surface–adsorbate interactions. The Atomic Simulation Environment (ASE)^43^ software was used to visualize crystal structures and postprocess the results.

## 3 Results and Discussion

The ternary WSSe nanotubes were grown using an atmospheric CVD reactor using WO_*x*_ whiskers, selenium, and H_2_S as precursors. As many as ∼50 reactions were conducted, which spanned the entire composition range from pure sulfur to (almost) pure selenium nanotubes. According to the SEM analyses, the yield of the nanotubes was very high on the surface of the agglomerates for *x*_Se_ = Se/(Se + S) < 0.7 and was somewhat smaller for the product highly enriched with respect to selenium. This trend is similar to the one reported in the previous work,^36^ in which closed ampules were used for the synthesis of such nanotubes. Unlike the previous work, however, pure WSe_2_ nanotubes could not be obtained in the flow reactor under the present conditions.

### 3.1 SEM and EDS Analyses

Since the top layer in the reactor crucible is in direct contact with the chalcogen vapors and acts as a diffusion barrier, the sulfurization/selenization proceeded quickly there, converting the nanowhiskers into flakes. Hence, no nanotubes were observed in L1 (Layer-1) for any composition of *x*_Se_ (A–E). WSSe nanotubes were abundant in the powder beneath layer L1 (layers L2–L6). In the fourth layer (L4) and lower, the sulfur and especially the selenium were scarce, and much oxide was left unreacted after the 30 min reaction. Figure 1(b,c) shows SEM images of a product prepared via the flow reactor from the E-2 batch. The low-magnification image (Figure 1b) shows that the product is heavily agglomerated, which is consistent with the oxide precursor powder (see Figure S1). The existence of heavily agglomerated nanoparticles complicates the interpretation of the analysis. Notably, SEM views the morphology of the features on the top surface only (a few nanometers), while the information depth of the energy dispersive spectroscopy (EDS) can be >1 μm. It is evident from the higher-magnification image (Figure 1b), that the yield of the nanotubes on the agglomerate surface is quite high, especially in the high-sulfur-containing phases (A–D), notwithstanding the little effort invested in optimizing the reaction yield in the present study. Figure S4a shows a high-magnification image of one such agglomerate (batch E-2), rich in nanotubes on its surface (Figure S4b). Statistical analysis of the diameter of more than 4000 nanotubes from batches B–E was carried out and is presented in Table S1. Batch F was excluded because the nanotubes were short and thick (Figure S5). Irrespective of the selenium/sulfur ratio, the diameters of the majority of the nanotubes were smaller than 40 nm. The nanotubes in the sulfur-rich composition ranged from 2 to 10 μm in length with a median length of 5 μm. The results of this analysis are not very surprising and reflect the fact that they are all produced from W_18_O_49_ nanowhiskers with similar average diameters and lengths.

Next, SEM-EDS analysis was performed in the center of several agglomerates of each layer. The analysis was performed for the different phases (A–F) and for each phase in the different layers (L2–L6). The results were averaged on different agglomerates and at different locations of the same agglomerate. Separately, SEM-EDS analysis was carried out on the periphery of several agglomerates from each batch, which consisted exclusively of nanotubes. The results of this analysis for layer 2 (L2) of the different compositions (A–F) are presented in Table 1. Further, Table S2a,b includes EDS information from other layers. Here O is the oxygen concentration (in atom %) divided by the sum of concentrations (in atom %) of oxygen, tungsten, sulfur, and selenium together, i.e., O/(W + O + S + Se) and similarly W is W/(W + O + S + Se).

Significantly, the diffusion of the chalcogen vapors is slower the deeper the layer is in the crucible. Hence, the O/W and the O/(S + Se) ratios increase the deeper the layer is in a given composition. For example, the O/W ratio is 0.28 for E-2 (in Table S2b) and 0.73 for E-3, which is one layer beneath E-2. Similarly, the ratio of O/(S + Se) for E2 is 0.22 and 0.42 for E3. The same trend is observed by comparing layers D2 and D4 and F2 and F3 layers. In fact, the difference in oxygen content of F2 and F3 (the Se-rich phase) is larger than in phases D and E. Therefore, one can conclude that the selenium diffusivity and reactivity with respect to the tungsten oxide crystallites are slower than that of sulfur. The increase in the O/W and O/(S + Se) ratios for deeper layers is attributed to the greater difficulty of both the sulfur and, more so, the selenium vapors to diffuse into the deeper layers and react with the tungsten oxide crystallites. In the same way, one notices also that *x*_Se_ is reduced from 0.51 in E-2 to 0.48 in E-3 in Table S2b, which is another manifestation of the fact that the selenium diffusion into the oxide precursor is slower the deeper the layer is in the crucible. Such differences are also observed in the periphery of the agglomerates, which are made solely of nanotubes (Table S2a). For instance, the O/W ratio is 1.01 for D2 and 1.98 for D4, which means that the oxide core of the nanotubes is on average larger the deeper the layers. Similarly, the O/(S + Se) ratio is 1.04 for D2 and 3.36 for D4.

**Figure 2:**
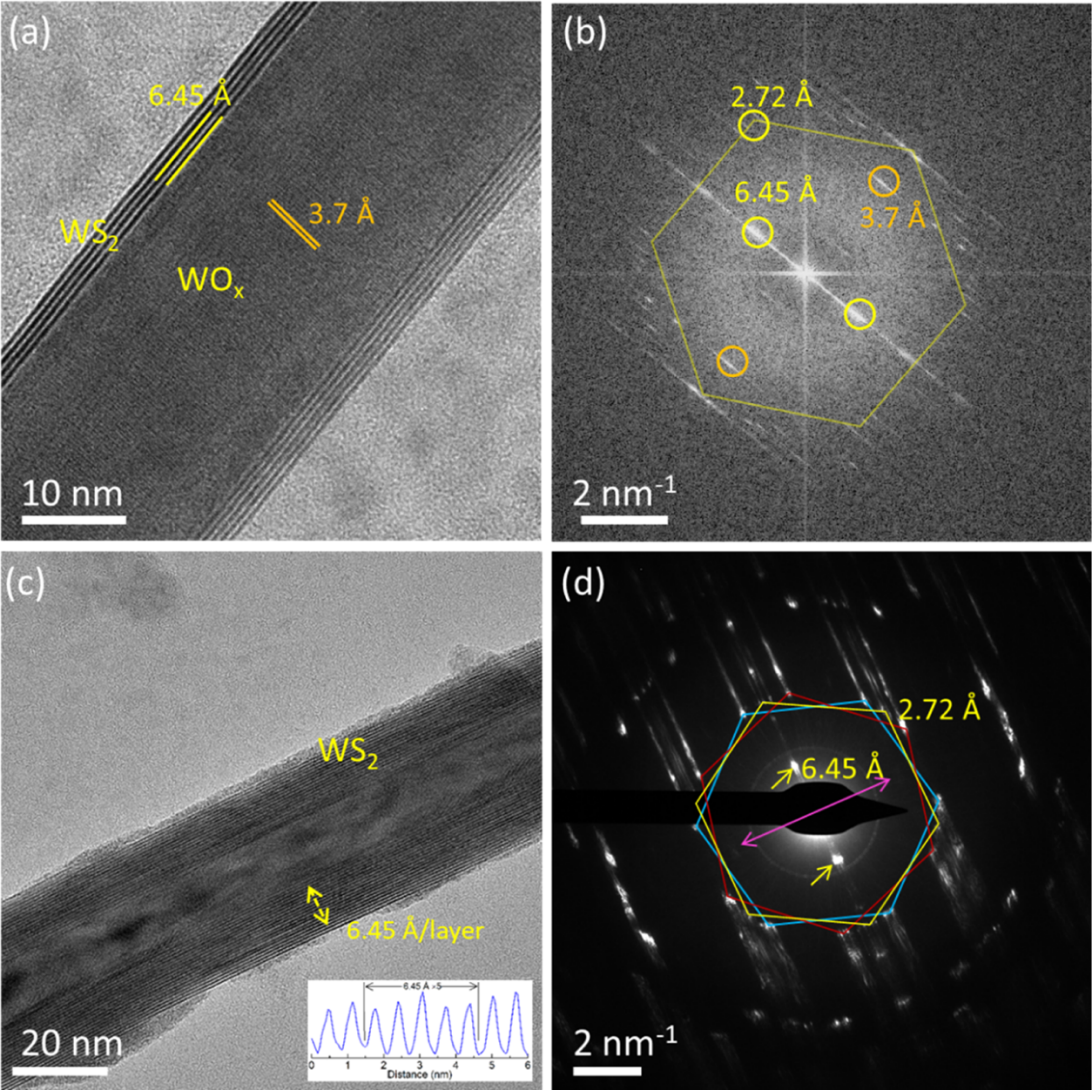
High-resolution (HR)-TEM image and corresponding Fourier transformation (FT) image of a WSSe-WO_*x*_ core–shell 1D nanostructure (a, b). *d*-spacings corresponding to core oxide and shell WSSe layers are shown. (c, d) HRTEM image and electron diffraction pattern of a hollow WSSe nanotube from E-2 composition *x*_Se_ = 0.5. The spacing between the layers is marked in the TEM image, and the corresponding intensity profile is shown in the inset (c). The pink double-headed arrow in the diffraction image corresponds to the nanotube growth direction. The interplanar spacings along the (002) and (100) planes of WSSe were marked. The hexagonal diffraction pattern corresponds to the 2H-WSSe polymorph, the rotation between the three hexagons indicates the different chirality, and the measured chiral angle is 11.3°.

The reaction mechanism and the kinetics of sulfurization/selenization are entirely different in the case of the nanowhiskers at the periphery of the agglomerate and the large oxide crystallites, which are abundant in the agglomerate core. In essence, after a few closed WSSe layers are formed on the surface of the nanowhiskers, the diffusion of sulfur and more so the selenium through the already closed WSSe basal (001) layers is suppressed considerably, leaving a large oxide core in the center of the nanotube (see also the TEM image in Figure 2) after half an hour reaction.^9,11^ In contrast, the diffusion of the chalcogen gases into the large tungsten oxide crystallites in the agglomerate center is rather fast, and its reactivity is high. Here, facile diffusion of the sulfur and the selenium atoms through the [100] (prismatic) direction of the large crystallites prevails, and hence the oxide gets reduced (and converted to the respective chalcogenide), faster. Therefore, in contrast to the oxide nanowhiskers in the periphery of the agglomerate, the large oxide crystallites in the agglomerate center were converted into WSSe flakes swiftly. This point is clearly demonstrated in Table 1, for example, where the O/(S + Se) ratio is 1.04 for layer D2 in the periphery (nanotubes) and is 0.49 in the core of the agglomerate, where large WSSe flakes are formed. This point is also made clear from the ratio of the O/W ratio. For example, the ratio O/W in E-2 is 1.64 for the periphery (nanotubes) and is only 0.68 for the core of the agglomerates where copious amounts of WSSe flakes are formed (Table 1). This trend is also confirmed for the deeper layers of the reaction product. Indeed, there is a prominent difference between the O/(S + Se) ratio in the center of the agglomerate (1.18 in layer D4 in Table S2b) and the bristles (core–shell nanotubes) in the periphery (3.36 in layer D4 in Table S2a). In conclusion, although the diffusion of the chalcogen gas into the agglomerate center is slower than its surface, its diffusion in the microscopic crystallites and reactivity are higher. Hence, the lower oxygen content in the center of the agglomerates can be attributed to the abundance of oxide microcrystallites, which react faster with the chalcogen gas converting into WSSe flakes. This fact is also surprising in view of the ∼100 times larger (normalized) surface area of the nanowhiskers compared to the micron-size crystallites in the center of the agglomerate.

Upon reduction of the flow of H_2_S gas and increase of the temperature of the selenium source, flakes and tubes enriched with selenium became predominant. The selenium-rich nanotubes were thicker, shorter, and somewhat less abundant than those formed in a sulfur-rich atmosphere (see Figure S5). The kinetics of the oxygen to chalcogen exchange is slowed down in the highly enriched selenium atmosphere. Therefore, in the selenium-rich phases (E and F) the WSSe skin of the core–shell nanotube is rather thin and the remaining oxide core is thick compared to the sulfur-rich phases (A–D). Although the O/(S + Se) ratio does not increase uniformly from A to F (see Tables 1 and S2a), overall, the oxygen content increases on going from the sulfur-rich (A) to selenium-rich (F) phases.

Since selenium permeates slower than sulfur, the ratio of selenium to sulfur in the product was found to decrease along the crucible depth (from L1 to L6). This trend is displayed in Figures S6 and S7, which show the selenium-to-sulfur concentration ratio in the center of the agglomerate and the nanotubes, respectively, as a function of their ratio in the vapor phase. Obviously, the deeper the layer in the boat, the lesser the selenium content in the product and specifically also in the nanotubes. Moreover, pure selenium lumps were observed in the product for reactions carried out in vapors highly enriched with respect to selenium, apart from the WSSe nanotubes and flakes. Consequently, one can conclude that the sulfur is chemically much more reactive toward the oxide nanowhiskers than the selenium. The difference in reactivity between the sulfur and the selenium can be attributed to the higher ionicity of the former, which reacts more readily with the ionic W–O bond of the WO_2.72_ nanowhiskers.

In summary, the SEM and SEM-EDS analyses allow one to draw already four important conclusions: (1) Notwithstanding the large difference in surface area, the high-temperature reaction of the W_18_O_49_ nanowhiskers with respect to the chalcogen vapor is appreciably slower than for large oxide crystallites. (2) The nanotube concentration decreases with increasing selenium content in the reaction mixture, especially in the selenium-enriched atmosphere; (3) The remaining suboxide W_18_O_49_ core^17,18,39^ in the nanotubes after half an hour reaction is larger when the selenium content is higher in the reaction mixture. These conclusions have important repercussions on the nanotubes’ structural, optical, and other properties, as reported below; (4) Lumps of free selenium were obtained in selenium-rich reactions along with nanotubes and flakes.

### 3.2 Transmission Electron Microscopy (TEM)

Extensive TEM analysis of the reaction products in the A–D phases was carried out. This analysis clearly revealed that the reaction was completed, and hollow nanotubes were obtained for about 50% of the tested objects. As expected, the sulfurization/selenization proceeded from the surface of the nanowhiskers inward, resulting in either hollow nanotubes or an intermediate product i.e., WO_*x*_@WSSe core–shell 1D nanostructures. Figure 2a,b shows HRTEM images and the corresponding Fourier transform (FT) of the core–shell structure comprising five closed WSSe layers, which crystallized in the 2H phase around the WO_*x*_ core. Although no detailed statistical analysis of the nanotubes’ diameter was carried out, it was found that the majority of the analyzed nanotubes possessed an average diameter below or close to 40 nm. The hexagonal diffraction pattern in the FT image corresponds to the WSSe layers, and the bright spots marked by the orange circle are from the (010) plane of the WO_*x*_. The chirality of the WSSe layers is clearly visible. The elongated electron diffraction reflections indicate the bending of the layers.^2^ The intermediate core–shell compound indicates that the reaction proceeded through the diffusion of sulfur/selenium and consumption of the WO_*x*_ template. The FT image shows that the *c*-axis of the nanotubes is perpendicular to the *b*-axis of WO_2.72_ whiskers, which signifies that chalcogenation indeed starts in the direction perpendicular to the nanowhisker growth axis and proceeds from the surface inward. The HRTEM image corresponding to electron diffraction (ED) in Figure 2c,d shows a WSSe nanotube without any oxide core from the E-2 batch. The ED pattern indicates that the lattice polymorph is a hexagonal 2H structure and exhibits a chiral angle of 11.3°.

**Figure 3:**
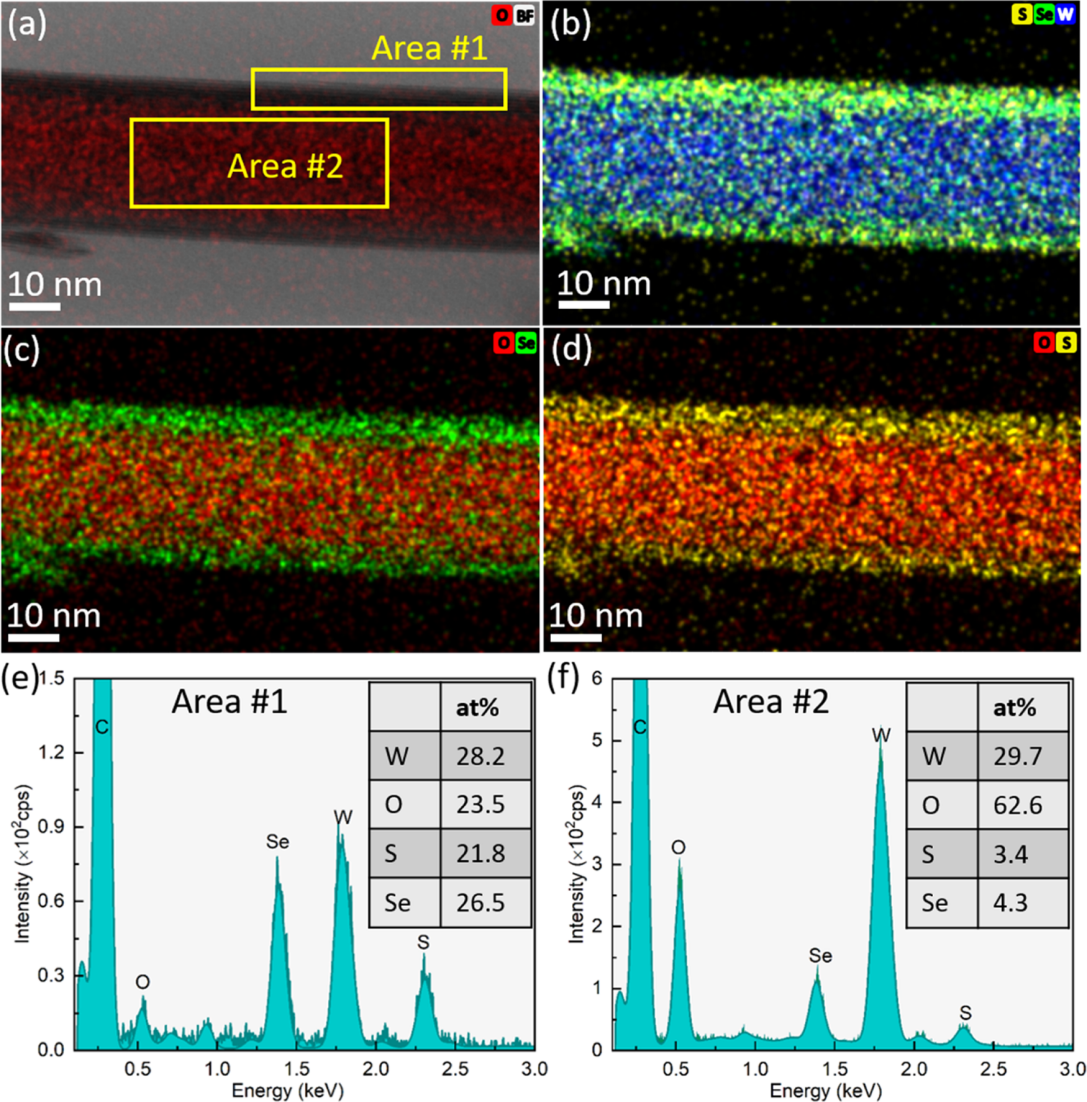
(a) Bright-field (BF) TEM image of a WSe_2*x*_S_2(1–*x*)_ nanotube (belonging to the E-2 phase) with an oxide core. The oxygen EDS map is overlaid on the TEM image showing a high concentration of oxygen in the core. (b–d) Overlapped EDS chemical maps of W–S–Se, Se–O, and S–O, respectively. (e, f) EDS spectrum collected from the different areas on the nanotube as marked in (a); the inset table indicates the quantification of the respective elements.

Figure 3 shows a TEM image of a WSe_2*x*_S_2(1–*x*)_ nanotube (E-2) and its EDS mapping, displaying a thick oxide core. Figure S8 also shows the scanning TEM–high-angle annular dark-field (STEM-HAADF) images of two nanotubes from the same batch, E-2. A hollow tube without oxide core (Figure S8a) and a core–shell structure (Figure S8b) with thick oxide core. Figure S9 shows the high-resolution STEM-HAADF image of another nanotube belonging to this series. The local structure of the WS_2_ layers is as anticipated 2H. Note the wavy appearance of the layers, which was attributed to the strain induced by the random sulfur and selenium distribution in the anion sublattice.^36^

EDS analysis of individual nanotubes in STEM mode (Figure 3) also showed that the oxygen content in the nanotube core is rather high, especially for the selenium-rich phases, which is ascribed to the short reaction time (half an hour). In general, this short reaction time was insufficient to convert the entire oxide core of all of the nanotubes into hollow nanotubes. However, the smaller the diameter of the nanotubes was, the more likely they were fully converted into hollow tubes. Qualitatively, about 50% of the nanotubes <40 nm had hollow core. Obviously, the tungsten oxide core can be transformed into tungsten sulfide-selenide upon further annealing. As stated above, the EDS analysis indicated that the concentration of the oxygen in individual nanotubes (the size of the oxide core) was larger, the higher the partial vapor pressure of selenium in the precursor. Figure 3e,f shows the elemental composition of the nanotube belonging to the E-2 batch. The Se/S ratio in area #1 is 1.22 (*x*_Se_ = 0.54) and is 1.26 (*x*_Se_ = 0.55) in area #2. This value is somewhat larger than the one cited in Table S2b, with 25.8/24.7 = 1.04 and 16.8/14.7 = 1.14 (for the center and periphery of the agglomerate of phase E-2, respectively). Therefore, there is only a qualitative agreement between the values reported by SEM-EDS for an assortment of nanotubes and those reported for individual nanotubes using TEM-EDS analysis. Obviously, the SEM-EDS data, which is based on extensive chemical and statistical analyses, is more representative than the values obtained through TEM-EDS of an individual nanotube.

### 3.3 DFT Calculations

To elucidate the formation of the whiskers, the following mechanism is hypothesized. First (step A), H_2_ molecules react with the O atoms on the surface to generate H_2_O vapor, leaving an oxygen vacancy on the surface
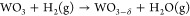
2

**Figure 4:**
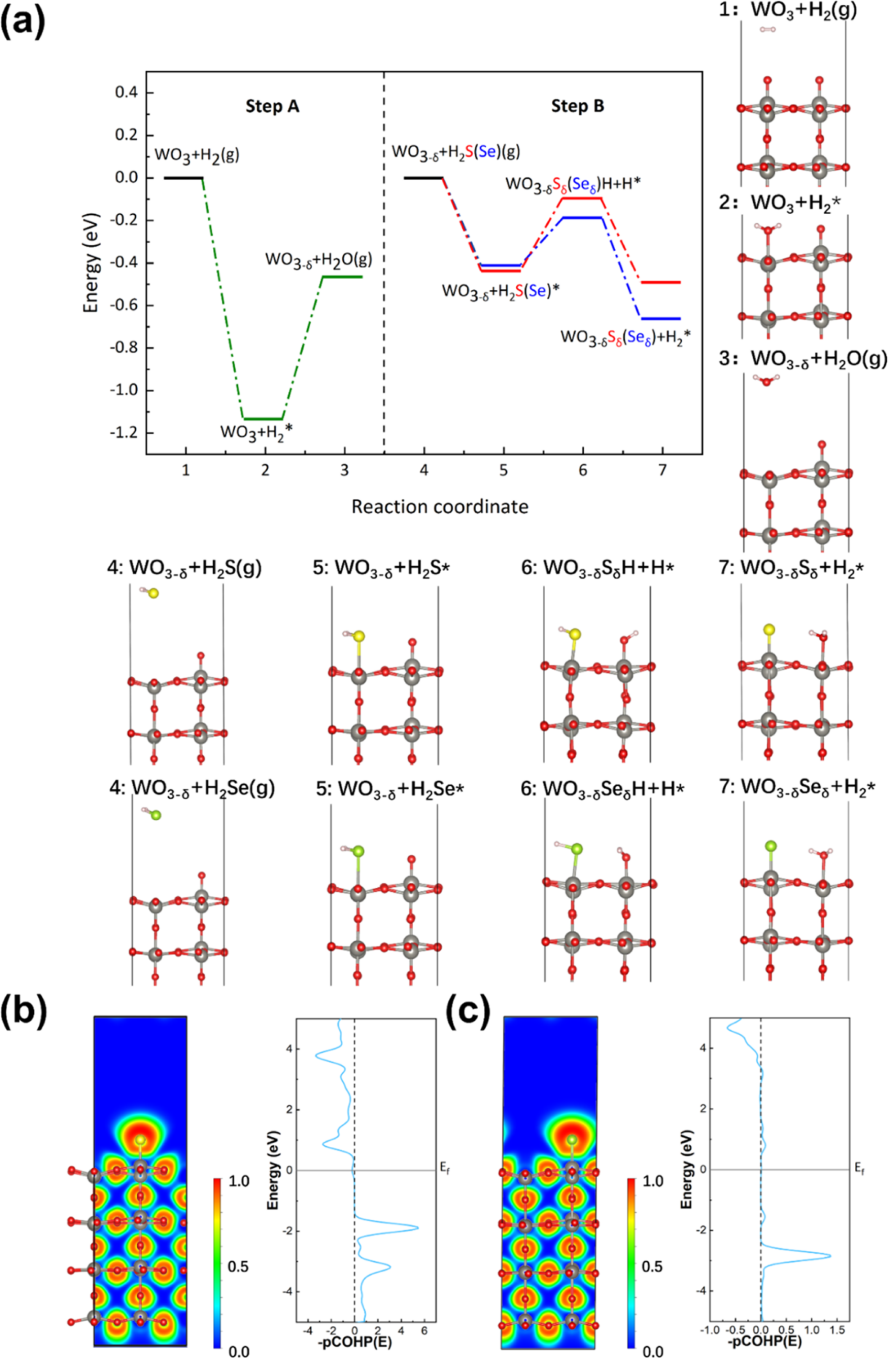
(a) Energy diagrams and corresponding adsorption configurations of H_2_ and H_2_S(Se) adsorbed on the WO_3_ (001) surface. ELF map and the COHP of (b) sulfur and (c) selenium adsorbed on WO_2.75_. Here, δ, *, and g represent an oxygen vacancy, chemical adsorption, and gaseous state on the WO_3_(001) surface. Gray, white, red, yellow, and green balls represent tungsten, hydrogen, oxygen, sulfur, and selenium atoms, respectively. One represents a covalent bond, whereas 0 represents no chemical bond formed in the ELF map.

Second (step B), H_2_S/Se is adsorbed on the surface, filling up the oxygen vacancy. Afterward, H_2_ desorbs either as H_2_ (g) or where each H atom migrates to the neighboring O, starting step B
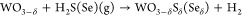
3The reaction pathway of step A on WO_3_(001) is shown in Figure 4a (left): when H_2_ absorbs on the surface of O atom, the total energy of the system is reduced by −1.13 eV, which means that this reaction can proceed spontaneously from the thermodynamic viewpoint. After the above reaction, the WO_3_ will form the component WO_2.75_ flakes, which is very close to the composition of W_18_O_49_ identified in the experiment. To study the reaction between WO_2.75_ flakes and H_2_S(Se) (step B), H_2_S or (H_2_Se) are adsorbed on WO_2.75_ as illustrated in Figure 4a. The H_2_S (or H_2_Se) vapor reacts spontaneously with WO_2.75_ allowing sulfur and selenium to chemically bond with W (reaction coordinate 4 → 5). Two paths were then followed. Each H of the H_2_S complex migrates to the neighboring O atom (reaction coordinate 5 → 7) and the reaction mechanism proceeds from step 1. Note that the splitting of H_2_S (H_2_Se) requires −0.49 eV for sulfur and −0.67 eV for selenium. Another possible pathway is that H_2_ simply desorbs from the surface as a molecule. This process is, however, very energetic and requires 2.49 eV for H_2_S and 1.52 eV for H_2_Se, suggesting a significant infeasibility of this reaction pathway. The calculations of the adsorption of individual S and Se on WO_2.75_ nanowhiskers found that S–W and Se–W chemical bonds form with energy releases of −1.39 and −0.94 eV, which indicates that the reaction is easier on sulfur (very truculent) than selenium sites (sluggish) as observed in the experiment. In addition, the difference between these two reactions from the perspective of chemical bonds by electron localization functional (ELF) and crystal orbital Hamilton population (COHP) is also demonstrated in Figure 4b,c. Both sulfur and selenium form ionic bonds with WO_2.75_ nanowhiskers, but sulfur exhibits stronger chemical bonds than selenium, which agrees well with the values of adsorption energy.

### 3.4 X-ray Powder Diffraction (XRD)

**Figure 5:**
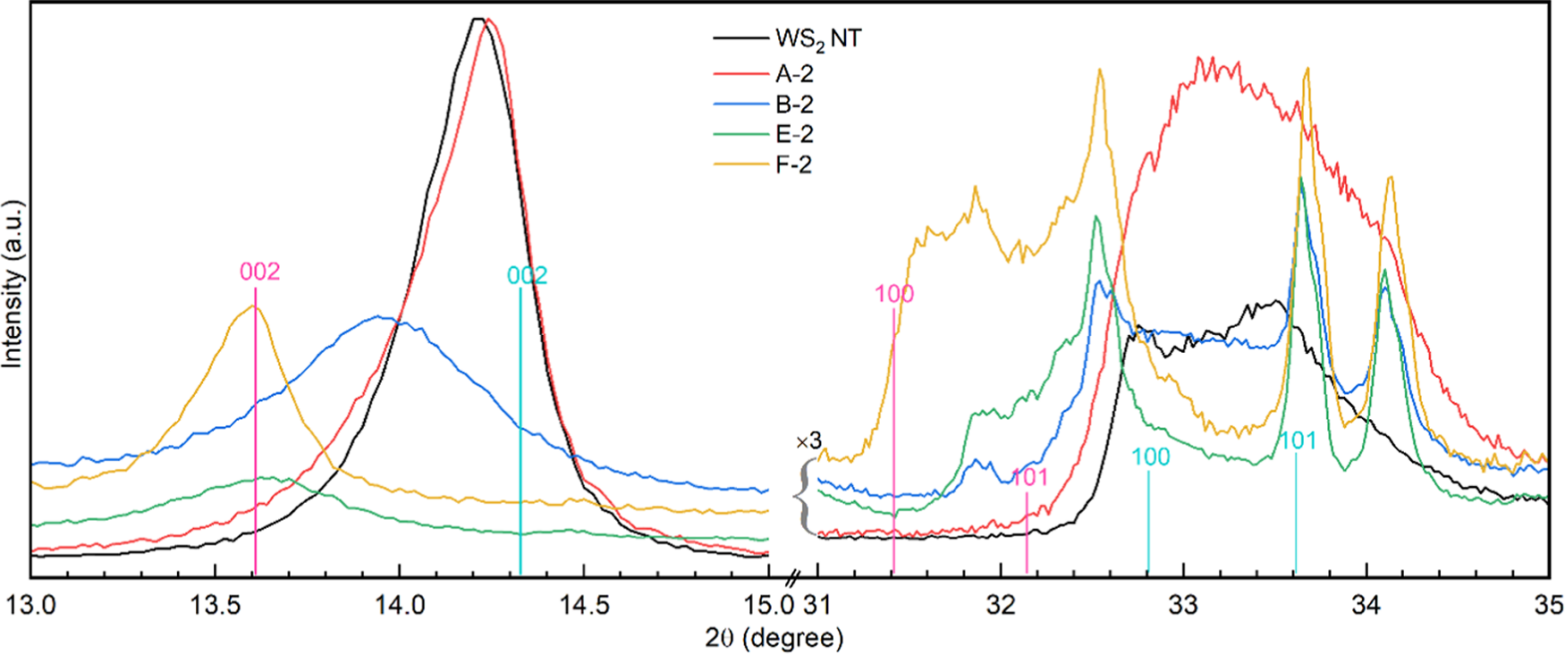
Extended view of the (002), (100), and (101) sets of XRD reflections measured for WSSe samples with ascending selenium content (A → F). The reflections indicated in cyan and pink correspond to the pure 2H phases of WS_2_ and WSe_2_, respectively.

Figure 5 displays enlarged XRD patterns of a few WS_2(1–*x*)_Se_2*x*_ phases with different *x*_Se_ near the (002), (100), and (110) peaks for the same phases (A–F). It must be kept in mind that the information collected here is from a depth of a few micrometers, i.e., beneath the surface of the agglomerated nanoparticles, which is not visible in an electron microscope (Figure 1). The XRD patterns are discussed qualitatively first. The (002) peak of the sulfur-rich phase (A-2) is similar to that of pure WS_2_ nanotubes but is asymmetric toward the low angles due to minute amounts of the selenium in this phase (see Table 1). Both the (002) and (100) peaks shift toward smaller angles with increasing selenium content in the product due to the larger size of the selenium atom compared to the sulfur atom, which leads to an increase of all of the *d*-spacings. When the selenium content in the phase increases, the (002) peak not only shifts to the left but also becomes wider and less intense, which can be attributed to a reduction in the WSSe “skin” thereby leaving a thicker unreacted oxide core (as shown by TEM in Figures 2 and 3). However, in the selenium-rich phase (F-2), the peak position almost coincides with that of 2H-WSe_2_; its intensity increases, and it becomes narrower.

The XRD pattern in the range of 31–34.5° contains contributions from both WSe_2*x*_S_2(1–*x*)_ and the tungsten oxide phases. In fact, the peaks at 32.6, 33.6, and 34.2° can be assigned to the W_18_O_49_ crystallites. The nanotubes obtained in the reaction with highly enriched selenium (>40 at%) vapor contained a large oxide core, which contributed the most to these peaks. In any event, according to their XRD patterns, the sulfur-rich products and pure WS_2_ nanotubes show little oxide content.

The asymmetry of the (100) and (101) peaks of WS_2_ nanotubes between 32 and 34° was discussed in previous works. Essentially, the stacking disorder^44^ of the layers due to their curvature and chirality leads to asymmetry of these X-ray diffraction peaks.^45^ Therefore, the degree of asymmetry in this peak provides a hint of the existence of nanotubes in the product. Indeed, as shown in Figure 5, the (100) peak at 32.7° is highly asymmetric in the pure WS_2_ nanotube phase. As the product becomes enriched with respect to selenium, the peak shifts to lower angles, becoming narrower and more symmetric. However, these profiles are more complex due to the presence of oxide peaks. Nevertheless, this trend hints at an increased abundance of flakes at the expense of nanotubes in selenium-enriched phases.

To put this analysis on a more quantitative basis, the shift of the (002) and (100) peaks due to the presence of nanotubes and their selenium to sulfur substitution must be estimated, first. Here one faces a dual problem. On the one hand, the (002) peak of nanotubes shifts to lower angles compared to that of the bulk 2H-WX_2_ (X = S, Se) due to the relaxation of the lattice strain in the curved layers of the nanotubes. However, the (100) peak hardly shifts for nanotubes compared to the bulk material.^16,19^ Replacing sulfur with selenium leads to a shift of these two peaks to lower angles, i.e., larger *d*-spacing. Table S3 summarizes the data and shows the peak shift due to both the formation of nanotubes and substitution of sulfur by selenium. Further, detailed analyses of the shifts in the XRD peak position and their correlation with standard flakes and nanotubes and the crystallite size analysis (Table S4) are given in the SI.

The XRD findings can be summarized by making references to three different processes that occur upon enrichment of the vapor with respect to selenium: (1) As the selenium concentration in the product increases, the (002) peak shifts to lower angles, i.e., larger interlayer spacing. The asymmetric (100) peak shows a similar shift but at a much slower rate. (2) The gradual decrease in the (002) peak intensity with increasing selenium content and its increased full width at half-maximum (FWHM) is attributed to the reduced “skin thickness” of the nanotubes. i.e., the product contains lesser amounts of WSSe due to the slower diffusivity of Se and much of the oxide content in the core remains unreacted. (3) Beyond say *x*_Se_ = 0.6 of selenium in the solid (D and F phases), the relative density of the nanotubes gets smaller and the majority of the product consists of 2H-WSe_2*x*_S_2(1–*x*)_ platelets. Since the platelets grow from the oxide phase faster than the nanotubes, the crystallite size grows; the oxide content goes down. Consequently, the (002) peak of the WSe_2*x*_S_2(1–*x*)_ phase (in F-2,3) becomes relatively stronger and narrower.

The individual nanotubes and flakes of the different series were characterized also via Raman spectroscopy, the results of which are discussed in the SI and are presented in Figure S10.

### 3.5 Extinction and Absorption Spectroscopy

Recently, the optical spectra of WS_2_ nanotubes with different diameters were studied.^24,46,47^ Major differences between the extinction and pure absorption spectra of nanotubes with a large diameter were found. Contrarily, nanotubes with diameter smaller than 60 nm exhibited similar extinction and absorption spectra. Careful analysis of the extinction spectra showed that owing to their large refractive index (>4), nanotubes with a diameter larger than 80 nm can confine optical modes (cavity modes) in their core. These cavity modes interact with the excitons exhibiting strong optical coupling and producing light-matter quasi-particles the so-called exciton-polaritons. The peak of the lowest polariton in the extinction spectrum is red-shifted (ca. 670 nm) compared to that of the A exciton (630 nm) in the absorption spectrum. On the other hand, a clear spectral dip appears in the extinction spectrum of the nanotubes near the wavelength of the A exciton peak in the absorption spectrum. The strong coupling phenomenon is manifested also by strong light scattering of the large-diameter nanotubes. Owing to opto-geometrical constraints, nanotubes with a smaller diameter cannot confine optical cavity modes in their core and their extinction and absorption spectra are similar.

Clear delineation between nanotubes, which exhibit a strong coupling effect (diameter >80 nm) and those which are not (diameter <60 nm) was vindicated through different optical measurements and simulation. Although the extinction was measured by simply collecting the light behind the sample, the intrinsic absorption of the nanotubes was measured with the help of an integrated sphere technique.^46^ Surprisingly, nanotubes that exhibit a strong coupling effect scatter light very effectively although their diameter (>80 nm) is 3–5 times smaller than the incident wavelength. Furthermore, the extinction spectrum of such nanotubes was found to be red-shifted compared to the net absorption measured via integrating sphere. A pronounced dip occurs in the extinction spectrum, which almost coincides with the A exciton peak position. This is one of the clear hallmarks of strong coupling with the A exciton. The two newly generated peaks, adjacent to the dip are the two hybridized energy states. These two peaks are considered as lower and upper polaritons. Also, the line shape in short wavelengths of the extinction spectrum was found to be entirely different from that of the absorption for such nanotubes with strong decay at short wavelengths (due possibly to strong Rayleigh scattering). On the other hand, the net absorption showed a gradual increase with decreasing wavelength, which reflects the increasing density of states at higher energies.

**Figure 6:**
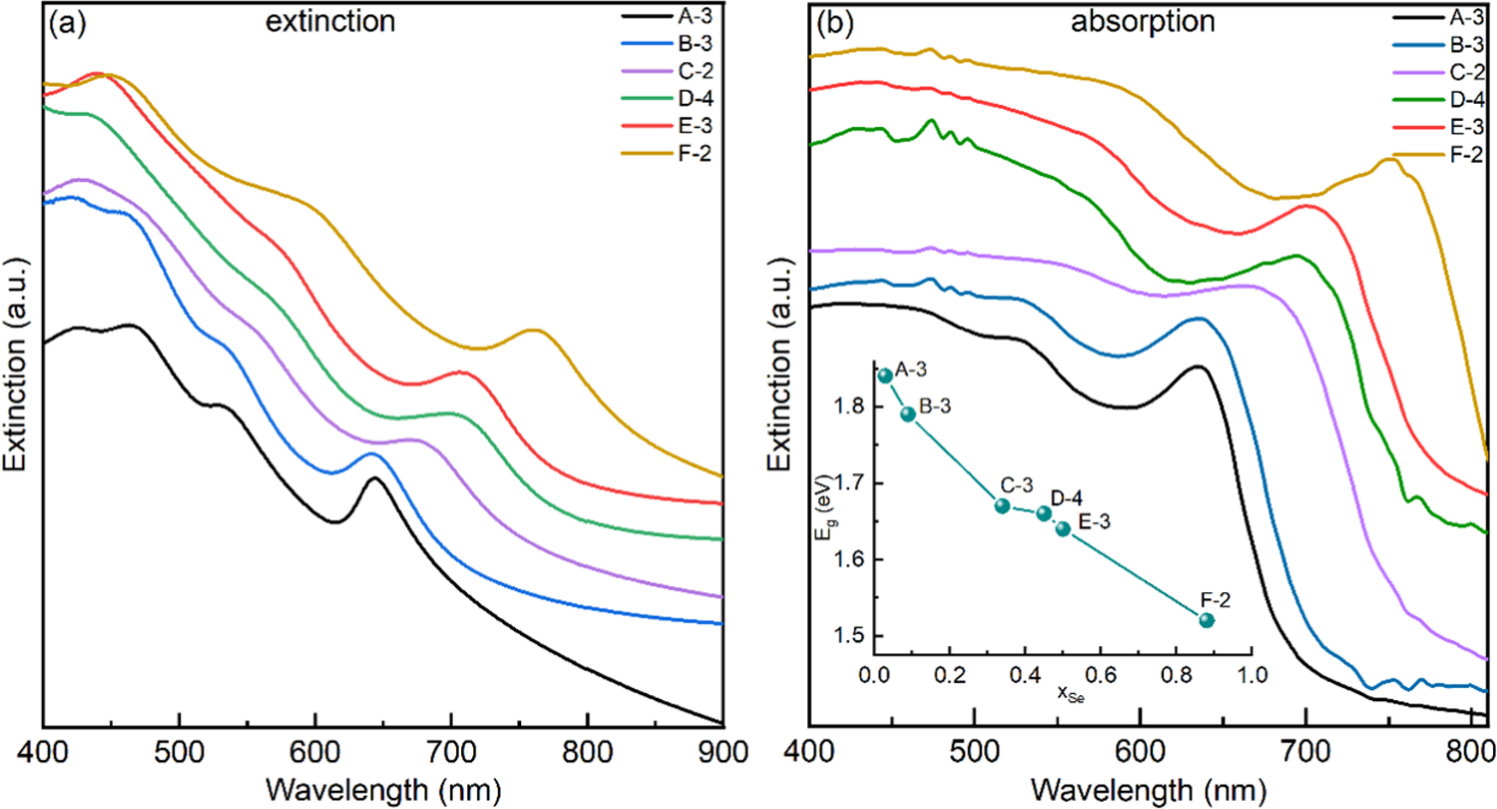
Comparison of extinction (a) and net absorption (b) features of WSe_2*x*_S_2(1–*x*)_ nanotubes with different Se compositions. Extinction was recorded by placing the sample in between the source and detector, whereas absorption was collected using an integrated sphere. The inset in (b) shows the dependence of the bandgap on the compositions obtained via the Tauc plots.

WSe_2*x*_S_2(1–*x*)_ nanotubes produced by the closed ampule technique also showed a strong coupling effect.^36^Figure 6 shows the extinction and absorption measurements of the different WSe_2*x*_S_2(1–*x*)_ nanotubes synthesized in this work. While Figure 6a shows the extinction measurements, Figure 6b shows the net absorption of the same samples using an integrating sphere setup. In contrast to the WSe_2*x*_S_2(1–*x*)_ nanotubes produced in the closed ampules,^36^ here the difference between the α peak position in the extinction measurements (Figure 6a) is small (see Table S5) compared to the position of the A exciton position in the absorption measurements (Figure 6b). Also, instead of the typical intensity decay with decreasing wavelength, as observed in the extinction spectra of WSe_2*x*_S_2(1–*x*)_ nanotubes produced by the closed ampule technique,^36^ here the intensity increases with decreasing wavelength. Figure S11 clearly demonstrates the differences between the WSe_2*x*_S_2(1–*x*)_ nanotubes produced by the closed ampule technique (green and orange curves) and the present nanotubes (red and black curves). More importantly, there is a significant difference between the line shape and the position of the peaks of the currently reported nanotubes compared to those produced by the closed ampule technique. The extinction of the nanotubes reported here increases with decreasing wavelength, which reflects the increasing density of states and enhanced absorption. However, the first peak of the nanotubes produced by the closed ampule technique is red-shifted with respect to the A extinction peak^36^ and the line shape of their extinction spectrum exhibits a decline below 650 nm. It can therefore be safely stated that the nanotubes produced via the closed ampule technique exhibit a strong coupling effect, while the present WSSe nanotubes seem to exhibit purely excitonic absorption. Obviously, the existence of flakes in the product may influence the spectral behavior of the product. First, the flakes have excess selenium compared to the nanotubes. Also, in general, the flakes do not exhibit a strong coupling effect. However, since these measurements were done in solutions, the large flakes precipitate first in the solution, leaving a nanotube-rich aqueous dispersion for the optical measurements.

In trying to understand the difference between the optical characteristics of WSe_2*x*_S_2(1–*x*)_ nanotubes produced by the closed ampule technique and those reported here, one should bear in mind two important differences between the two kinds of nanotubes. First, one should recall that the two kinds of nanotubes were produced by sulfurization-selenization of W_18_O_49_ nanowhiskers of different batches. Apparently, the average diameter of the oxide nanowhiskers used in the previous work was higher. Consequently, the average diameter of the tubes obtained by the closed ampule method was 70–80 nm while it is 40–50 nm here. Apart from being narrower (see Table S1), the present nanotubes contained large oxide core, which can be estimated to occupy some 2/3 of their volume. While the refractive index of the sulfide-selenide shell is >4, that of the oxide is ∼2.^48^ The refractive index of the core–shell WO_3–*x*_@WSSe nanotubes reported here can be grossly averaged arithmetically to 2.5–2.8. Therefore, such core–shell nanotubes were unable to confine the light and produce cavity modes, reducing the possibility of a strong coupling effect here.

Using the Tauc plot,^48^ the direct gap transitions of the WSSe nanotubes could be estimated as a function of the composition (*x*_Se_). The results are summarized in the inset of Figure 6b, where the (almost) linear tunability of the bandgap with composition is demonstrated. The quantitative values obtained by this analysis are nevertheless questionable, since the direct bandgap calculated from the Tauc plots is smaller by more than 100 meV with respect to the energy of the A excitonic transition for each composition. Note that the present analysis agrees with the optical bandgaps calculated from the onset of the electron energy loss spectra (EELS) of WSSe nanotubes obtained via the closed ampule method (Figures 4 and 5 in ref (36)). These values are 100 meV smaller than the values of the A exciton of the nanotubes of the same composition as determined by both EELS and the values determined from optical data.^36^ One would anticipate though that the exciton energy is below the bandgap and not higher as reported here. This puzzle must be further investigated in future studies.

Finally, the transient absorption spectra behave entirely differently for the two families of nanotubes.^24,46,49^ The exciton cavity-mode coupling interactions in the nanotubes are probed through transient absorption (in fact transient extinction) measurements (Figure S12). While the nanotubes with a large diameter (produced by the closed ampule technique) exhibit significant spectral diffusion to the blue as a function of the delay time, those with small diameters synthesized here show only small spectral shift with the delay time. Thus, the TA spectra show no spectral diffusivity of the presently synthesized nanotubes and hence the lack of a strong coupling effect, which is consistent with the extinction and absorption measurements.

## 4 Conclusions

The synthesis of WSe_2*x*_S_2(1–*x*)_ nanotubes is appreciably more demanding and has been discussed succinctly in the literature. WSe_2*x*_S_2(1–*x*)_ nanotubes with 0 ≤ *x* < 1 were synthesized in this work using a flow reactor, where W_18_O_49_ nanowhiskers, H_2_S gas, and selenium vapors served as the precursors and under reducing atmosphere at 840 °C. In contrast to the preceding work where similar nanotubes were synthesized in closed ampules,^36^ the present technique is amenable for scaling up in the future, as was previously done for pure WS_2_ nanotubes.^19^ Practically, since the reaction was carried out for a short time only (30 min), the reaction was not completed and some 50% of the nanotubes (particularly those with large diameter and enriched with respect to selenium) showed a remnant tungsten oxide core. This observation was attributed to the larger size and perhaps also its smaller ionicity of the selenium atom compared to sulfur. Ab initio computations confirmed the energetic favorability of the oxygen extraction from the tungsten oxide and, subsequently, the oxygen to sulfur/selenium exchange of the reaction pathway proposed in this study. The structure and composition of the hollow nanotubes and core–shell WO_3–*x*_@WSSe nanotubes were thoroughly characterized via several techniques. Most of the nanotubes were found to have diameters smaller than 40 nm. The selenium and sulfur atoms were found to be randomly distributed on the anion lattice site. The bandgap and excitonic transitions were found to be linearly modulated with the composition.

## Supporting Information Available

Characterization details, electron microscopy images, X-ray diffraction, Raman spectra of WSSe nanotubes, steady-state extinction, absorption, and pump–probe optical studies (PDF)
