# Supplementary material for: Insights into the Growth of Ternary WSSe Nanotubes in an Atmospheric CVD Reactor
Source: Inorg Chem. 2023 Oct 24;62(44):18267–79. doi: 10.1021/acs.inorgchem.3c02903 (PMC10630937; doi:10.1021/acs.inorgchem.3c02903)
Supplement: Supplementary file 1 — ic3c02903_si_001.pdf [file ic3c02903_si_001.pdf]

Supporting Information for

Insights into the Growth of Ternary WSSe Nanotubes in an Atmospheric *CVD* Reactor

R. Rosentsveig,[1#] M. B. Sreedhara,[1,2#*] S. S. Sinha,[3] I. Kaplan-Ashiri,[4] O. Brontvein,[4] Y. Feldman,[4] I. Pinkas,[4] K. Zheng,[5] I. E. Castelli[5*] and R. Tenne[1*]

[1]Department of Molecular Chemistry and Materials Science, Weizmann Institute of Science, Rehovot 7610001, Israel
[2]Solid State and Structural Chemistry Unit, Indian Institute of Science, Bengaluru 560012, India
[3]Plasmon Nanotechnologies, Istituto Italiano Di Tecnologia, Via Morego 30, Genova, 16163, Italy
[4]Department of Chemical Research Support, Weizmann Institute of Science, Rehovot 7610001, Israel
[5]Department of Energy Conversion and Storage, Technical University of Denmark, DK-2800 Kgs. Lyngby, Denmark

[#]equal contribution

*corresponding authors email- reshef.tenne@weizmann.ac.il (RT); sreedhara@iisc.ac.in (MBS); ivca@dtu.dk (IEC)

SI Content

1. Materials Characterization

Scanning electron microscopy (SEM)

Transmission electron microscopy (TEM)

X-ray powder diffraction (XRD)

Raman spectroscopy

Steady-state extinction and absorption measurements

Pump-probe optical (transient absorption) measurements

2. SI figures and discussion

Figure S1 to S12
Table S1 to S5
XRD discussion
Raman analysis and discussion
Transient absorption analysis and discussion

1. Materials Characterization:

Scanning electron microscopy (SEM): Scanning electron microscopy (SEM) analysis was done with a Zeiss Sigma 500 model. A minute quantity of native sample was picked up by a capillary tube and dispersed on

carbon tape for the SEM analysis. Energy dispersive X-ray spectroscopy (EDS) analysis and mapping were performed using a retractable four quadrants detector (Bruker QUANTAX FlatQUAD) installed on a Zeiss Ultra 55 SEM. Hypermaps of the NTs and the agglomerates were acquired at 5kV for improved lateral resolution (the W intensity maps were plotted using its M lines). The quantification of the elements is based on a standard-less and self-calibrating spectrum analysis procedure using the ZAF matrix correction formulas.

Transmission electron microscopy (TEM): A Talos F200X G2 TEM/STEM 200 kV (Thermo Fisher Scientific, USA) was used for the preliminary analysis. A double Haider-Rose hexapole aberration-corrected Themis Z microscope (Thermo Fisher, USA) equipped with a high-brightness FEG at an accelerating voltage of 300 kV was employed for HRSTEM imaging. HAADF-STEM images were recorded with a Fischione Model 3000 detector with a semi-convergence angle of 30 mrad, a probe current of 40 pA, and an inner collection angle of 70 mrad. EDS hyperspectral maps were collected with a SuperX G2 four-segment SDD detector with a probe semi-convergence angle of 21 mrad, a beam current of approximately 200 pA, a pixel dwell time of 20 µs and a total recording time of typically 10 minutes. Quantitative maps were analyzed with the Velox software (Thermo Fisher, USA), through background subtraction and spectrum deconvolution by empirical model.

X-ray powder diffraction (XRD): X-ray powder diffraction (XRD) was performed using TTRAX III (Rigaku, Tokyo, Japan) theta-theta diffractometer. The setup was equipped with a rotating copper anode X-ray tube operating at 50 kV/200 mA and with a scintillation detector aligned at the diffracted beam after a bent Graphite monochromator. The powder samples were placed on a zero-background Si substrate. They were scanned in specular diffraction mode ($\vartheta/2\vartheta$ scans) from 4-70º (2ϑ) with a step size of 0.02º and a scan rate of 0.5º per min. Phase and profile analyses were made using the Jade Pro software (Materials Data, Inc.) and PDF-4+ 2022 database (ICDD).

Raman spectroscopy: A minute quantity of the sample was dispersed in ethanol by sonication and drop cast on a glass substrate. Raman scattering measurements in the range from 100 to 1000 cm^{-1} were recorded on individual NTs using the back-scattering mode. The measurements were carried out on a LabRAM HR Evolution spectrometer (HORIBA, France) equipped with different lasers. For the 633 nm laser, the maximum incident power on the sample was 0.225 mW. Given the spot size (1 µm) of the laser, the real incident power on the NT was ~ 0.02 mW. The LabRAM is fitted with an 800 mm spectrograph with a very high spectral resolution and low stray light. Frequency calibration was performed before every measurement session using a Si wafer, with a peak at 520.7 cm^{-1} of single-crystalline Si(100). Initial measurements were recorded with a 600 groves/mm grating with $\sim$1.8 cm^{-1} pixel resolution. Subsequently, for a detailed analysis, the measurements were done with 1800 groves/mm grating, with a pixel resolution of $\sim$0.35 cm^{-1}. The NTs were illuminated using several microscope objectives (MPlanFL NA=0.9, Olympus, Japan). The system utilizes an open confocal microscope (Olympus BXFM) with a spatial resolution better than 1 µm. Due to the very high aspect ratio of the NTs, it was very easy to visualize an individual NT with 100x or x150 objective and analyze it. The Raman spectra were collected in a 1024 × 256 pixel front illuminated open electrode CCD camera (Syncerity, HORIBA, USA), which was cooled to -60 °C.

Steady-state extinction and absorption measurements: UV–Vis extinction spectra were measured using a Cary-5000 UV-VIS-NIR spectrometer (Varian). In all the cases, a few milligrams of samples were dispersed in spectroscopic grade ethanol and ultra-sonicated for a few minutes to get the uniform dispersions. The

samples were quickly dispersible and the suspension was stable during measurement. All suspensions were measured in quartz cuvettes in the range of 1000 to 400 nm with 1 nm interval.

Integrating sphere (Hamamatsu Quantaurus QY) was used to measure the absolute absorbance of the dispersed $WSe_{2x}S_{2(1-x)}$ (0≤x≤1) nanotubes. The samples are placed inside an integrating sphere for the measurements. This instrument directly measures the amount of absorbed light in the sample, by placing the sample in the integrating sphere. The system was calibrated each time using a reference sample with known absorbance (quartz cuvette with pure ethanol to extract the net optical absorbance). A calibration for counting the single-pass absorption photons was performed to avoid full extinction, which also includes photons that are scattered a few times before reaching the detector.

Pump-probe optical (transient absorption) measurements: The transient absorption setup used for this study consisted of a 420 nm ultrashort pulse (roughly 120 fs pulse duration) of light produced by an amplified Ti:Sapphire system (Spitfire ACE, Mai Tai SP, Empower 45, Spectra-Physics, Santa Clara, CA) coupled to an OPA (TOPAS-Prime, and NIRUVIS harmonic generator, Light Conversion, Vilnius, Lithuania). Following the pump pulse, the sample was probed at varying delays by a white-light continuum from a CaF_2 window (with a spectral range of 370–750 nm). A filter was placed in the beam path to reduce the intensity of the fundamental beam (800 nm), covering the visible-near infrared (vis–NIR) region. The pump and probe beams cross and overlap within the cuvette holding the sample and the probe beam is collected into an optical fiber bringing it into a Triax 190 spectrograph (Horiba Jobin Yvon, France). The spectrograph is equipped with a Newton CCD camera (Andor, UK), which collects every pulse of the 1 kHz train of probe pulses from the laser system and calculates an absorption spectrum from two consecutive pulses of probe light (one with and one without the pump pulse which is removed by a synchronized mechanical chopper). The system utilizes delay lines (Aerotech, USA) for the pump and probe beams fitted with retroreflectors, allowing for delays of up to 4 ns, which is controlled by LabView software. The data was analyzed using SurfaceXplorer (Ultrafast Systems, Sarasota, FL). Transients were acquired with sub-picosecond resolution up to 4 ns.

2. SI figures and discussion

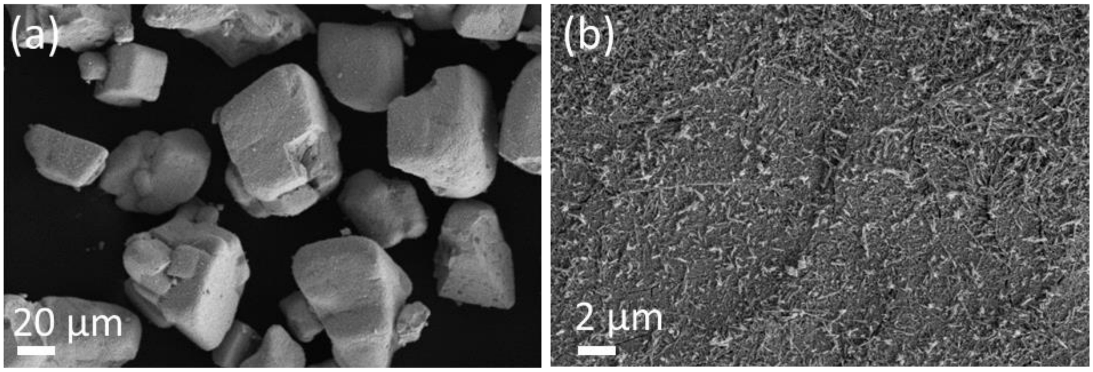

Fig. S1. SEM micrograph of the agglomerated $WO_{2.92}$ powder with $W_{18}O_{49}$ nanowhiskers in two magnifications.

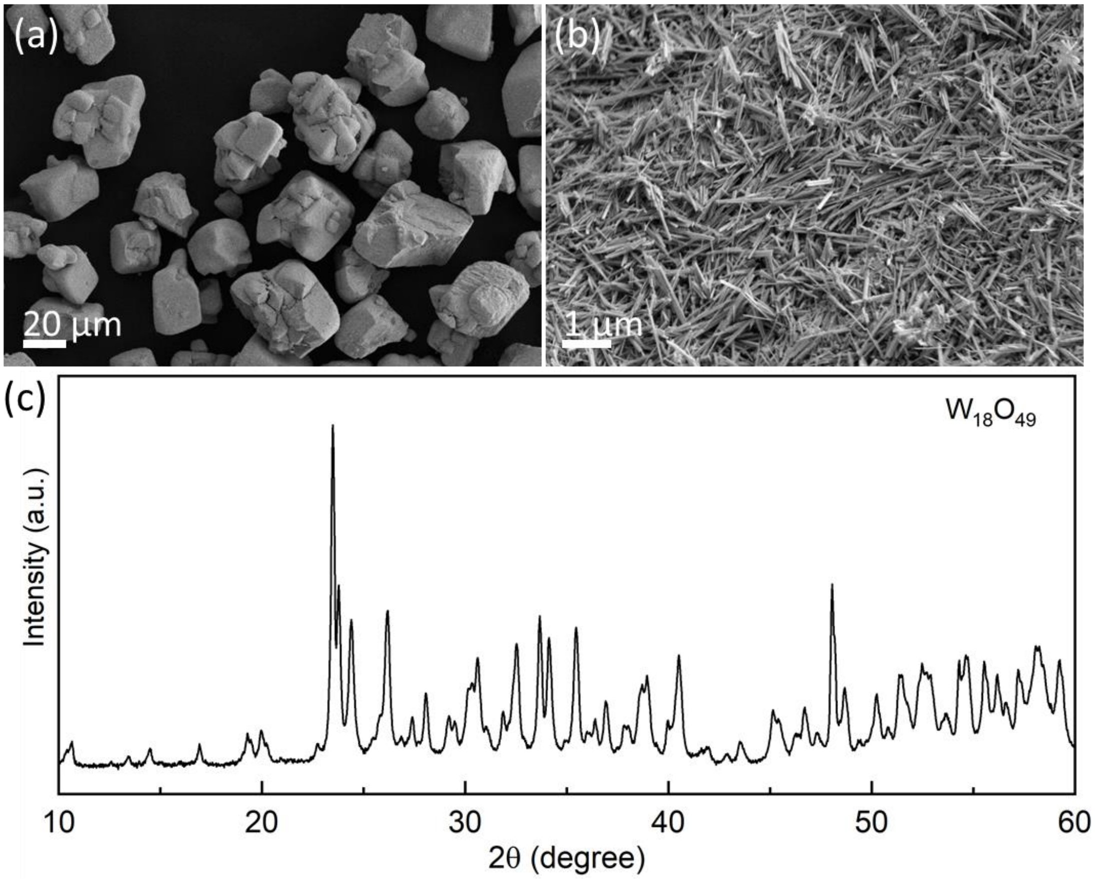

Fig. S2. (a) Low magnification SEM image of oxide nanoparticles after high-temperature treatment in hydrogen gas (b) high magnification image of the surface of the oxide particles showing the formation of $W_{18}O_{49}$ nanowhiskers (c) Powder XRD of the treated $WO_{2.92}$ oxide particles, which are perfectly matched with the $W_{18}O_{49}$ phase (PDF file number 04-007-5649)

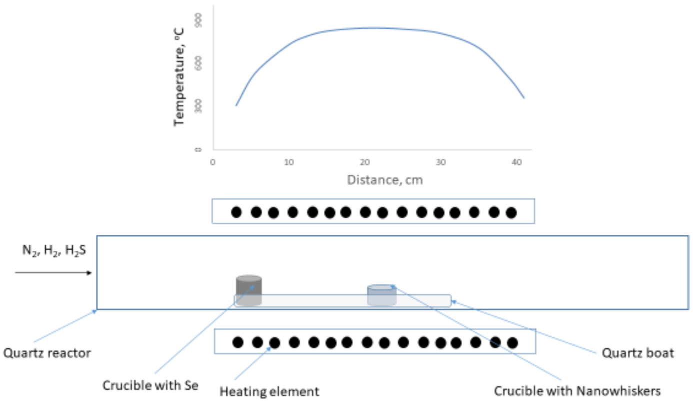

Fig. S3. Schematic rendering of the flow reactor and the temperature profile used in the present work.

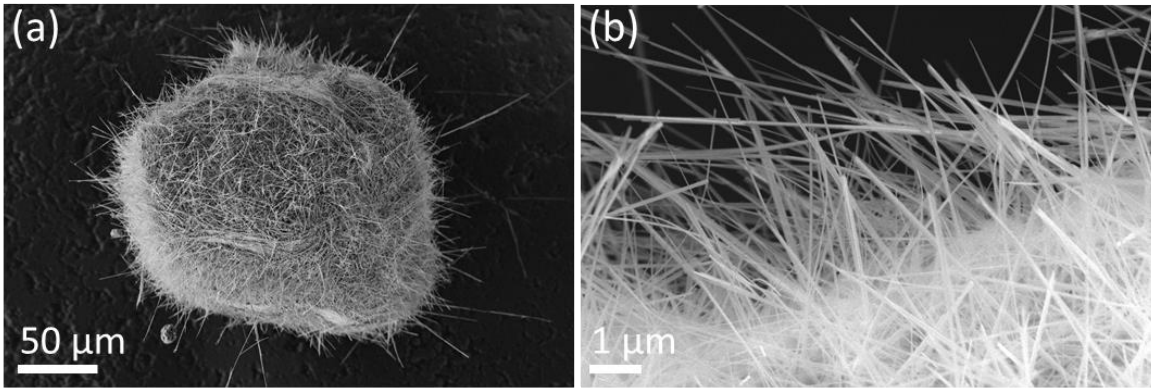

Fig. S4. (a-b) Low and high magnification SEM image of an agglomerate of the (E-2) batch with nanotubes protruding from its entire surface.

Table S1. Statistical analysis of the nanotube diameter from SEM images. In this analysis, a few thousand nanotubes from batches B, C, D and E were analyzed (A and F were excluded)

**Fraction of NT with diameter (D)**							
**Layer No.**	**D average (nm)**	**less than 33 nm**	**33<D<50**	**50<D<80**	**80<D<100**	**100<D<120**	**D>120**
L2	41	0.795	0.075	0.075	0.025	0.015	0.015
L3	35	0.840	0.095	0.040	0.015	0.005	0.005
L4	38	0.850	0.045	0.070	0.020	0.005	0.010
L6	37	0.800	0.100	0.080	0.015	0.005	0.000

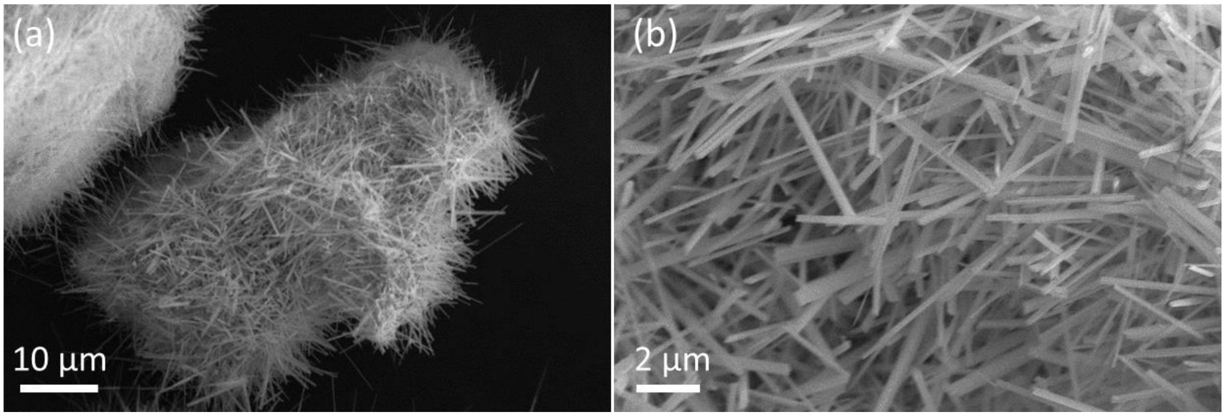

Fig. S5. (a) SEM image of an agglomerate from the **F-2** batch (Se rich phase) and its magnified image (b). The nanotubes here are thicker and shorter than those described in **Figs. 2** and **S3**, which contain less selenium. In some other areas of this batch, there were fewer nanotubes but they exhibited a larger aspect ratio.

Table S2. Composition of the agglomerates studied via SEM-EDS of the different compositions: (a) at the periphery of the agglomerate, where nanotubes protrude from its surface (b) and its center, see also **Fig. S3**.

(a) the periphery of the agglomerate							
**Table (a)**	**Se (at%)**	**S (at%)**	**W (at%)**	**O (at%)**	**O/W**	**x_{Se} @periphery**	**O/(S+Se)**
**A-2**	1.5	49.4	28.8	20.3	0.70	0.03	0.40
**B-2-3**	2.4	23.1	26.8	47.7	1.78	0.09	1.87
**C-2**	10.4	19.9	30.5	39.2	1.29	0.34	1.29
**D-2**	15.8	16.8	33.4	33.9	1.01	0.48	1.04
**D-4**	7.4	9.1	28.0	55.5	1.98	0.45	3.36
**E-2**	16.8	14.7	25.9	42.6	1.64	0.53	1.35
**E-3**	14.7	14.7	30.8	39.8	1.29	0.50	1.35
**F-2**	18.8	2.6	30.1	48.5	1.61	0.88	2.27
**F-3**	6.7	1.9	15.7	75.7	4.82	0.78	8.80
(b) at center of the agglomerate							
**Table (b)**	**Se (at%)**	**S(at%)**	**W(at%)**	**O(at%)**	**O/W**	**x_{Se} at centre**	**O/(S+Se)**
**A-3**	1.9	45.9	35.0	17.2	0.49	0.04	0.36
**B-2**	4.1	34.6	38.0	23.3	0.61	0.11	0.60
**C-2**	18.6	37.6	34.2	9.6	0.28	0.33	0.17
**C-4**	8.0	18.7	34.5	38.8	1.12	0.30	1.45
**D-2**	17.6	23.3	39.1	19.9	0.51	0.43	0.49
**D-4**	10.8	14.7	44.4	30.1	0.68	0.42	1.18
**E-2**	25.8	24.7	38.5	11.0	0.28	0.51	0.22
**E-3**	22.0	23.8	35.0	19.2	0.73	0.48	0.42
**F-2**	27.5	4.1	43.4	25.0	0.58	0.87	0.79
**F-3**	13.3	4.1	28.6	54.00	1.89	0.77	3.10

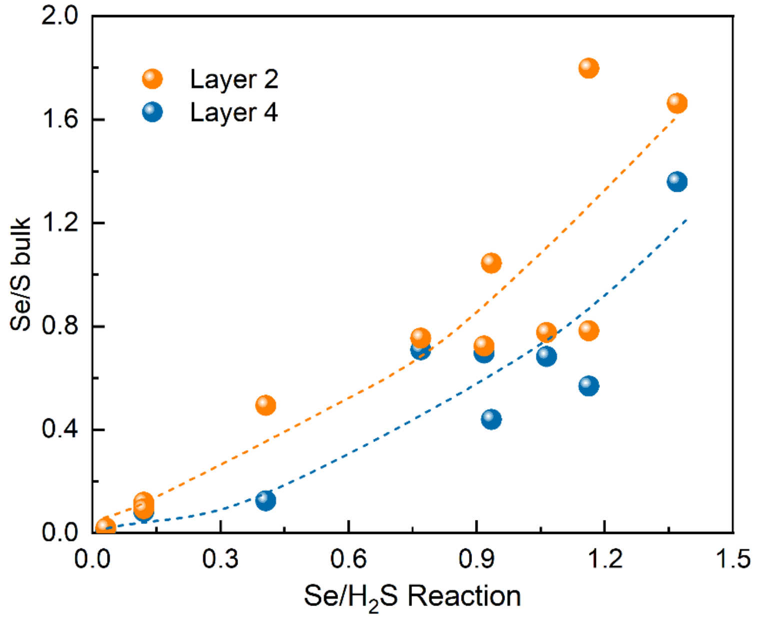

Fig. S6. The Se/S ratio in the product was obtained through SEM-EDS analysis of the agglomerates as a function of their ratio in the vapor phase (reaction precursors). The points are experimental, while the dashed lines were obtained by curve-fitting and serve as a guide to the eye. Note that the Se/S ratio goes down with distance from the top surface of the oxide precursor in the boat.

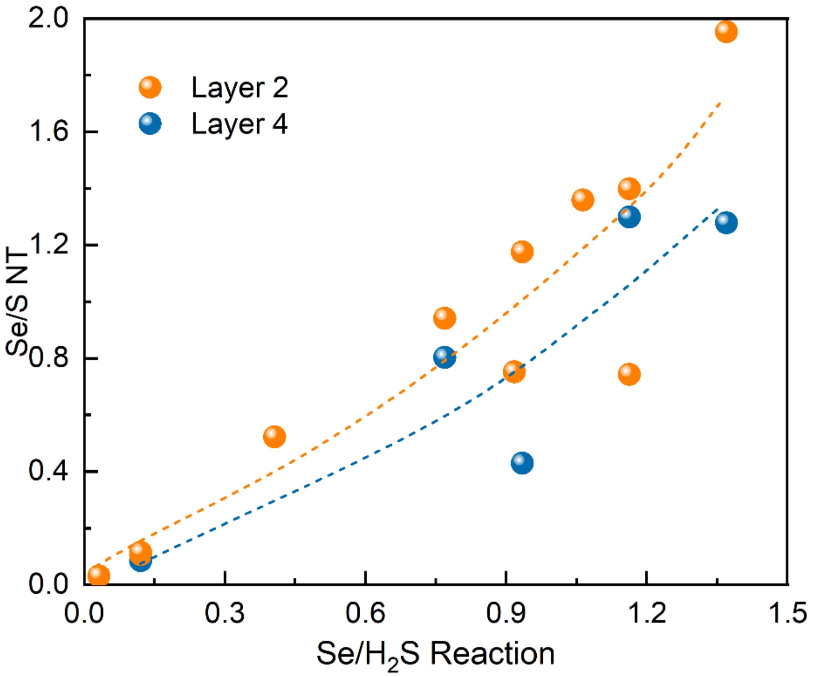

Fig. S7. The Se/S ratio in the WSSe nanotubes was obtained through SEM-EDS analysis of the periphery of the agglomerates as a function of their ratio in the vapor phase. The points are experimental, while the dashed lines were obtained by curve-fitting and serve as a guide to the eye. Note that the Se/S ratio goes down with distance from the top surface of the oxide precursor in the boat.

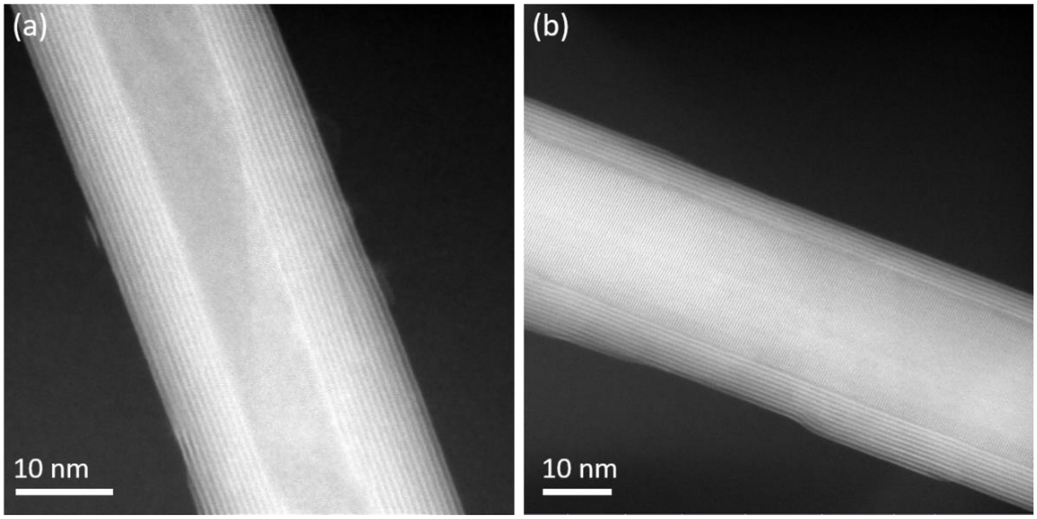

Fig. S8. STEM-HAADF images of tubes belonging to the E-2 batch with hollow (a) and oxide full core (b) in the center.

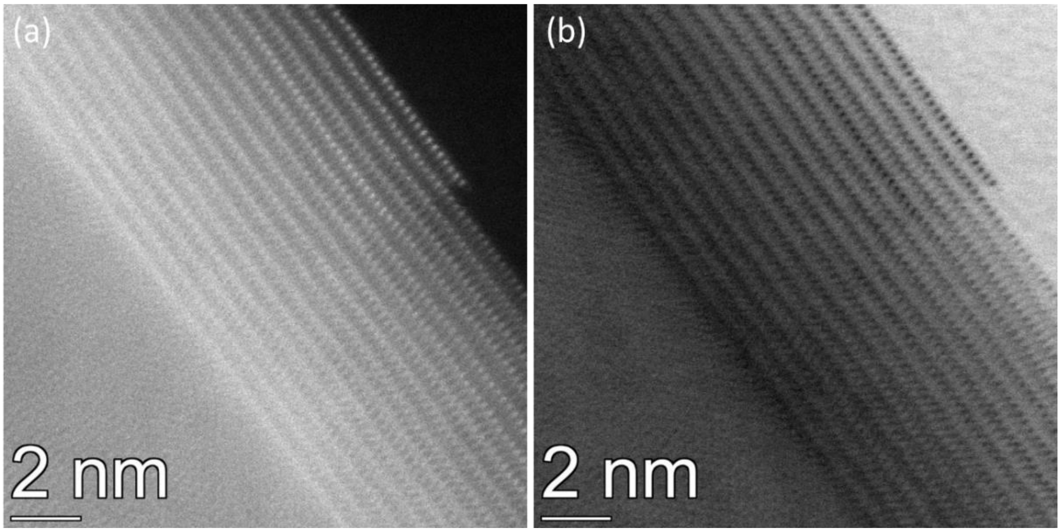

Fig. S9. High-resolution STEM and HAADF image of a WSSe nanotube with E-2 composition. The nanotube contains 15 layers of WSSe and the outer 16th layer is abruptly ending in the surface indicate the wrapping. (a) HAADF image; (b) BF image of the same area of the nanotube.

XRD analysis

To determine the position of the (002) peaks, a profile-fitting procedure was used. The total shift "Shift $\Delta d = d_{WS2(1\text{-}x)Se2x} – d_{2H\text{-}WS2}$" and the relative shift (in %) , i.e. "Relative Shift $\Delta d/d_{2H\text{-}WS2}$*100%") were calculated for the (002) peak, first. Unfortunately, this procedure is not applicable to the highly asymmetric (100) peaks. Therefore, in the next step, the shifts of the (100) peaks in the $WS_{2(1\text{-}x)}Se_{2x}$ phases is estimated by taking the midpoint in the left slopes of these peaks ("Shift $\Delta d = d_{WSe2xS2(1\text{-}x)} – d_{2H\text{-}WS2}$" in **Table S4**) and the relative peak shift of the (100) peak (in %), i.e. "Relative Shift $\Delta d/d_{2H\text{-}WS2}$*100%" is calculated. The shifts Δd of the (002) and (100) peaks in the $WSe_{2x}S_{2(1\text{-}x)}$ phases relative to their positions for bulk (2H) WS_2 are

shown. In the next step, the difference between the relative shifts of the two peaks, i.e. "Difference between relative shifts of (002) and (100)" was calculated. As can be seen from **Table S4**, the increment, i.e., "Relative Shift $\Delta d/d_{2H\text{-}WS2}*100\%$" in the interlayer spacing (002) in WSe_2 compared to WS_2 (5.1%) is larger than the increment in the (100) peak (4.3%) by 0.8% and is again 0.8% for WS_2 nanotubes (compared to bulk 2H-WS_2).

Comparing the "differences in the relative shifts of the (002) and (100) peaks" between the sulfur/selenium mixture samples and pure nanotube phase of WSe_2, one can make a conjecture that the ratio of nanotubes and flakes (platelets) decreases in selenium enriched $WSe_{2x}S_{2(1-x)}$ phases. It turns out that the relative shift of sample A-2, which contains very little selenium (0.6 at% in **Table S4**), is smaller than that of the pure nanotube phase (WS_2 NTs), i.e., 0.8%. Presumably, this difference attests to the fact that the A-2 sample also contains platelets, which are not strained. The same logic holds for the selenium-rich sample F-2, in which this parameter (1.8%) is lower than that of the pure nanotube phase of WSe_2 (2.1%), which indicates that this sample also contains flakes. Such estimates do not work for samples with intermediate selenium and sulfur content since they contain a relatively large amount of oxide, whose peaks influence the position of the chalcogenide peaks. Knowing that the (100) peaks hardly shift for the nanotubes, the peak shifts of (100) allowed estimating the Se content in the phase (**Table S4**, right column). This calculation turned out to be close to the estimates obtained from the EDS (**Table 1**).

Further insight into the growth of the nanotubes was obtained with the help of an analysis of the oxide content in the product in comparison to the chalcogenide content by the quantitative analysis of the entire XRD spectrum and the crystallite size (coherent scattering length) as calculated from the Scherer formula using the (002) peak. **Table S5** summarizes the data of this analysis.

First, the crystallite size in the [001] direction goes down and the oxide content increases as x_{Se} increases, which is attributed to the enrichment of the selenium content in the nanotubes, as discussed above. However, as the selenium fraction increases beyond x_{Se}= 0.6, the product is enriched with micron-size $WSe_{2x}S_{2(1-x)}$ platelets rather than nanotubes, and hence the crystallite size increases and the oxide concentration goes down. This analysis is consistent with the previous data.

Table S3. Comparison of the shifts of (002) and (100) XRD peaks.

	(002)			(100)			**Difference between relative shifts of (002) and (100) in %**	
**Sample**	***d*-spacing** Å	**Shift** $\Delta d = d_{WS2(1-x)Se2x} - d_{2H\text{-}WS2}$ Å	**Relative Shift** $\Delta d/d_{2H\text{-}WS2}*100$ %	***d*-spacing** Å	**Shift** $\Delta d = d_{WSe2xS2(1-x)} - d_{2H\text{-}WS2}$ Å	**Relative Shift** $\Delta d/d_{2H\text{-}WS2}*100$ %		x_{Se}
2H-WS_2	6.175	0	0	2.728	0	0		
2H-WSe_2	6.492	0.317	5.1	2.846	0.112	4.3	0.8	
WS_2 NTs	6.223	0.048	0.8		0	0	0.8	
WSe_2 NTs	6.556	0.381	6.2		0.111	4.1	2.1	
A-2	6.215	0.04	0.6		0	0	0.6	
B-2	6.348	0.173	2.8		0.016	0.6	2.2	0.14
E-2	6.499	0.324	5.2		0.067	2.5	2.7	0.58
F-2	6.506	0.331	5.4		0.097	3.6	1.8	0.84

Table S4. Composition and the domain size in [001] direction of the products calculated from XRD analysis.

**Sample**	**Approximate crystalline size of the $WSe_{2x}S_{2(1-x)}$ phase in the [001] direction(nm)**	**$WSe_{2x}S_{2(1-x)}$ content (at%)**	**Tungsten oxide content (at%)**
A-2	23	93	7
B-2	13	48	52
E-2	19	40	60
F-2	25	58	42

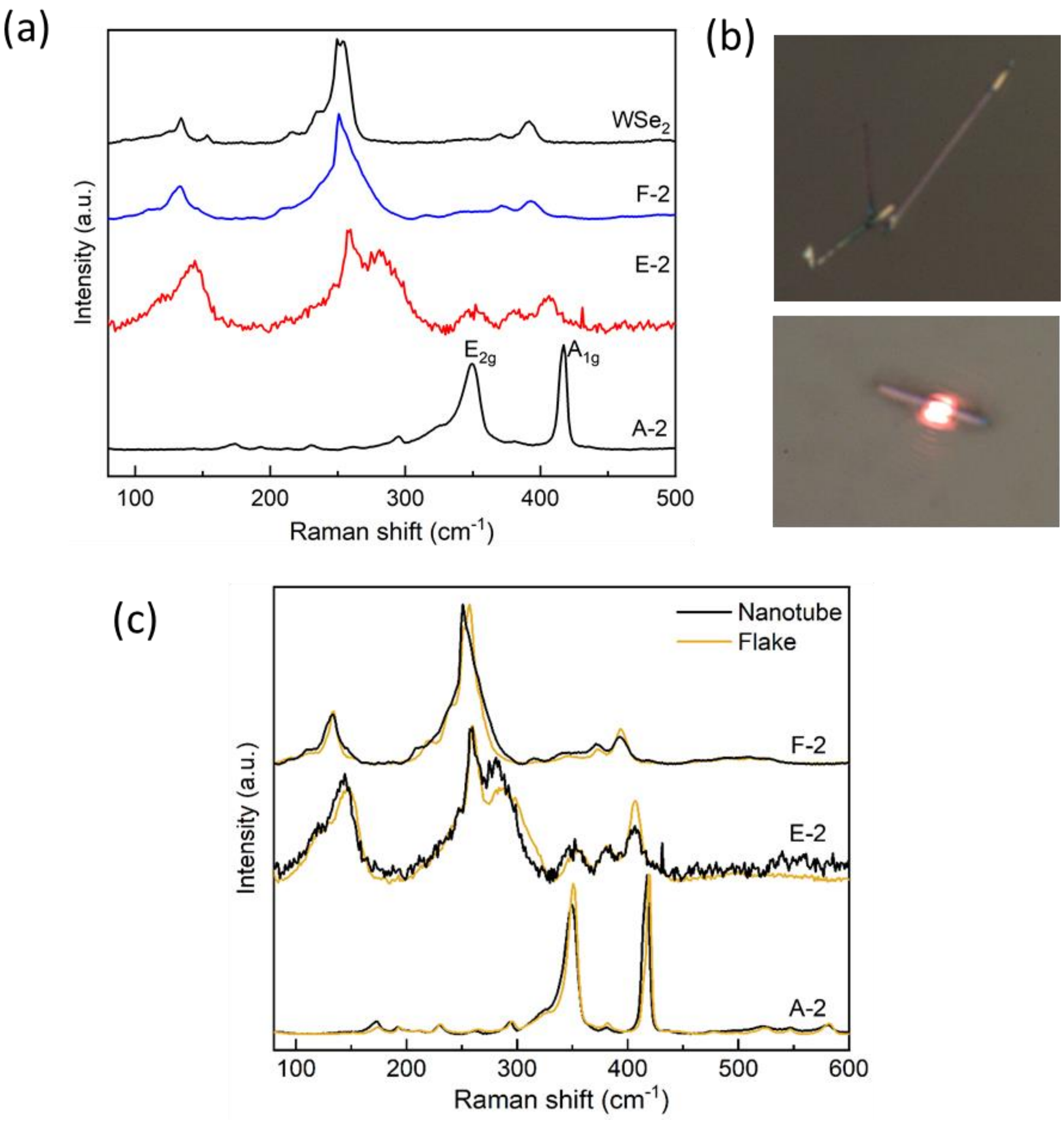

Fig. S10. (a) Raman spectra of several $WS_{2(1-x)}Se_{2x}$ nanotubes with different Se compositions collected using the 633 nm laser, which is in resonance with the WS_2 optical gap, the spectrum of a pure WSe_2 nanotube is taken from Ref. 32 (main text). (b) Representative optical images of the $WS_{2(1-x)}Se_{2x}$ nanotubes on which Raman spectrum has been collected (up), a 633 nm red laser focus on one such nanotube is shown (down). x_{Se} was calculated based on the global SEM-EDS data (**Table 1**); (c) Comparative Raman spectra of flakes and tubes belonging to different batches.

Raman analysis: Raman analysis of individual nanotubes of different compositions and flakes was undertaken. The Raman spectra of $WS_{2(1-x)}Se_{2x}$ flakes with varying selenium content (x_{Se}) was published before.[1, 2] The Raman spectrum of pure WS_2 nanotubes was studied before[3] and consists mainly of the E_{2g}^1 (shear) mode at 354 cm^{-1} and the A_{1g} (radial) mode at 423 cm^{-1}. In WSe_2 these two modes almost coalesce into a single broadened peak at 253 cm^{-1}.[4, 5] The excitation wavelength used here (633 nm) can be considered to be in resonance with the A exciton of WS_2 (622 nm) and the B-exciton of WSe_2 (636 nm). **Fig. S10** shows the Raman spectra of a few $WS_{2(1-x)}Se_{2x}$ nanotubes and pure WSe_2 nanotubes (taken from Ref. 4). As concerns **Fig. S10**, there were minor variation in Raman signatures between different tubes belonging to the same composition, but the picture is consistent overall with the one published before.[4] First, the Raman spectra of x_{Se}= 0.04 (A-2, see **Table 1)** nanotubes are quite similar to that of pure WS_2 nanotubes, with minor differences in the peak positions. The spectrum of the nanotube x_{Se}= 0.48 (E-2) is quite analogous to the spectra shown in **Fig. S8** in Ref. [4] between x_{Se}=0.42 and x_{Se}= 0.62 (Se/S = 1.33). The spectrum of an individual tube belonging to batch F-2 bears similarities to those of x_{Se}=0.77, i.e. Se/S = 3.35 and pure WSe_2 nanotubes. Given the concentration deviation from one batch to another and from one tube in the same batch to the other, the picture obtained from the Raman spectrum is quite consistent with the EDS and XRD analysis. Finally, **Fig. S10c** shows the Raman spectrum of an individual tube and a flake belonging to the A-2; E-2 and F-2 batches. The spectrum of the A-2 batch is not very different from that of pure WS_2. The E-2 flake has a strong A_{1g} peak at 405 cm^{-1}, which in fact, indicates that it is richer with respect to selenium than the nanotube. The spectra of the nanotube and flake belonging to the F-2 batch are quite similar, suggesting that their composition is similar, too. Therefore, the Raman analysis shows that the selenium content of the flakes is similar to that of the tubes or even smaller.

Table S5. Comparison table for the A exciton position obtained by extinction and absolute absorption measurements carried out in the integrated sphere. The similarity in the A exciton position infers that there is no strong coupling effect in the samples prepared in this study.

Sample	Extinction wavelength (nm)	Absorption wavelength (nm)
A-3	644	634
B-3	641	636
C-2	670	666
E-3	707	700
D-4	696	694
F-2	760	751

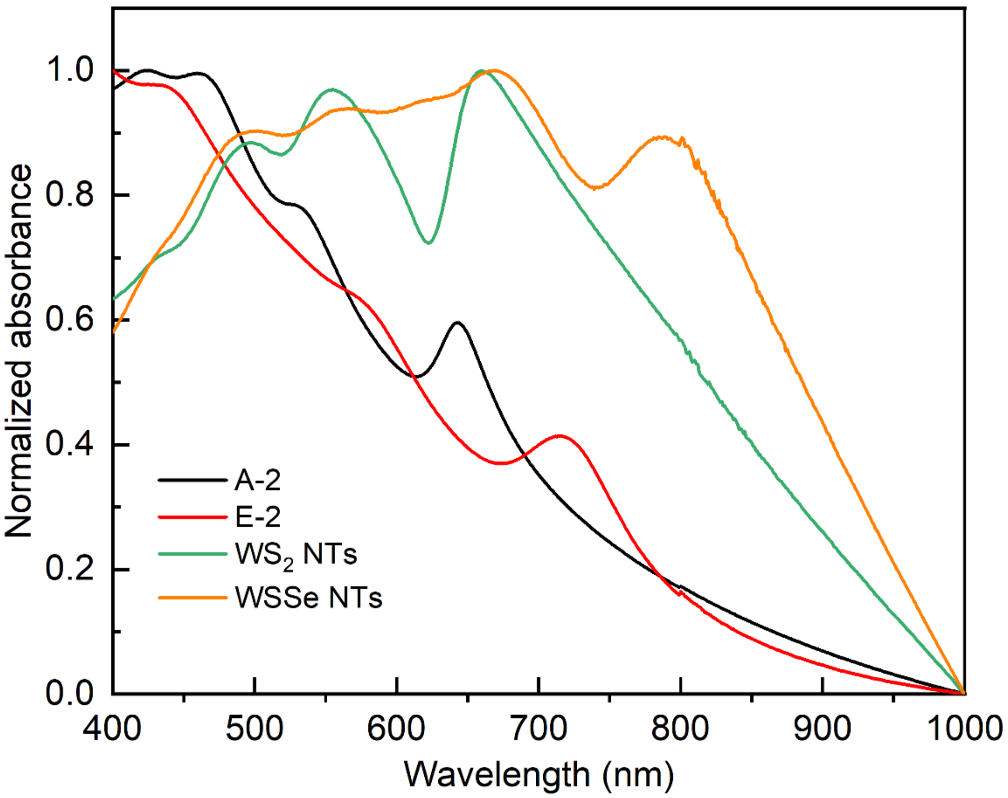

Fig. S11. Comparison of extinction graph of pure WS_2/WSSe nanotube with samples prepared in the present study with almost similar composition. The strong scattering of light can be seen in the case of nanotubes which is the hallmark of strong light-matter interaction and as a result forms polaritons. The features of the present sample are purely excitonic though they are measured in the same conditions. The polaritonic features were almost absent due to the small size of the nanotube or filled by an oxide core which does not confine light to form cavity modes.

Transient absorption: **Fig. S12** represents the transient absorption analysis of three different samples dispersed in water. The position of the excitons as a function of the selenium content is clearly realized from the absolute absorption measurements (**Fig. 7**b and **Table S6**). Unlike the previously reported measurements,[4, 6, 7] many of the (selenium-rich phases) nanotubes in these materials are not empty

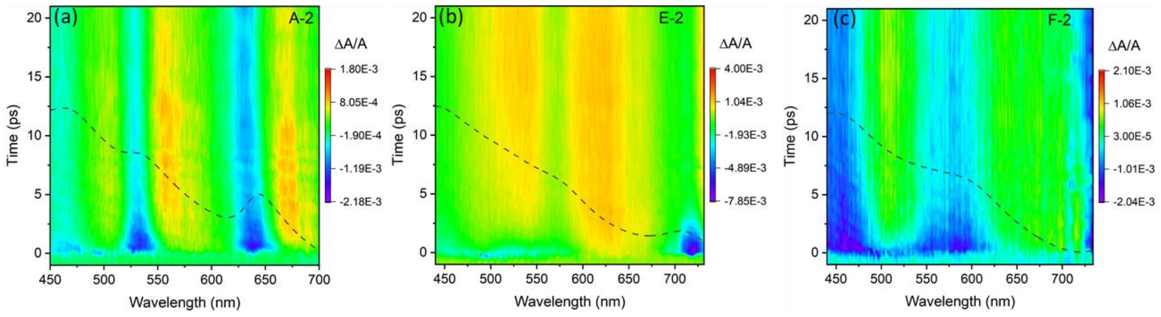

Fig. S12. Transient absorption spectra of (a) batch A-2, (b) E-2 and (c) F-2. The black dashed line in each contour indicates the extinction spectra of the samples. Note: The dynamics of the A exciton is observed in the contour plot (a) whereas the same for the B exciton in the contour plot (c).

containing oxide material (WO_3) instead of a hollow core. The refractive index of the core is ~ 2.[8] Due to the relatively low refractive index, a cavity leakage is observed instead of a stable standing optical mode. Hence, polariton formation is hindered due to the lack of exciton-cavity coupling.

This is further realized in the transient absorption experiments presented in **Fig. S12**. In the case of sample A-2, ultrafast dynamics of the A exciton is observed. Here, a minor spectral diffusion of the lowest energy dip with time is observed. This suggests[6, 9, 10] that, in the highly enriched sulfur phase, the majority of the product consists of nanotubes with possibly a small fraction of them being with a diameter of 80 nm and above. In the case of the other two samples, e.g. E-2 and F-2, the A excitons are shifted beyond 700 nm. Due to the filtering of the light source below 800 nm and the resultant poor signal-to-noise ratio, the dynamics of the A exciton could not be followed in this case. The dynamics of the B excitons are presented for these samples in **Fig. 12**b&c. Clearly, these features show little spectral diffusivity with time, indicating that the nanotubes are not making the majority of the phase in the product. In fact, since the refractive index of WSe_2 is larger than that of WS_2,[4] it would be anticipated that nanotubes as thin as 60 nm could maintain a cavity mode, but they do not seem to be there, at least in sufficient amount to influence the TA spectra.

References

1. Duan, X.; Wang, C.; Fan, Z.; Hao, G.; Kou, L.; Halim, U.; Li, H.; Wu, X.; Wang, Y.; Jiang, J.; Pan, A.; Huang, Y.; Yu, R.; Duan, X., Synthesis of $WS_{2x}Se_{2-2x}$ Alloy Nanosheets with Composition-Tunable Electronic Properties. *Nano Lett.* **2016,** 16, (1), 264-269.
2. Ko, K. Y.; Lee, S.; Park, K.; Kim, Y.; Woo, W. J.; Kim, D.; Song, J.-G.; Park, J.; Kim, J. H.; Lee, Z.; Kim, H., High-Performance Gas Sensor Using a Large-Area $WS_{2x}Se_{2-2x}$ Alloy for Low-Power Operation Wearable Applications. *ACS Appl. Mater. Interfaces* **2018,** 10, (40), 34163-34171.
3. Viršek, M.; Jesih, A.; Milošević, I.; Damnjanović, M.; Remškar, M., Raman scattering of the MoS_2 and WS_2 single nanotubes. *Surface science* **2007,** 601, (13), 2868-2872.
4. Sreedhara, M. B.; Miroshnikov, Y.; Zheng, K.; Houben, L.; Hettler, S.; Arenal, R.; Pinkas, I.; Sinha, S. S.; Castelli, I. E.; Tenne, R., Nanotubes from Ternary $WS_{2(1-x)}Se_{2x}$ Alloys: Stoichiometry Modulated Tunable Optical Properties. *J. Am. Chem. Soc.* **2022,** 144, (23), 10530-10542.
5. del Corro, E.; Terrones, H.; Elias, A.; Fantini, C.; Feng, S.; Nguyen, M. A.; Mallouk, T. E.; Terrones, M.; Pimenta, M. A., Excited Excitonic States in 1L, 2L, 3L, and Bulk WSe_2 Observed by Resonant Raman Spectroscopy. *ACS Nano* **2014,** 8, (9), 9629-9635.
6. Yadgarov, L.; Višić, B.; Abir, T.; Tenne, R.; Polyakov, A. Y.; Levi, R.; Dolgova, T. V.; Zubyuk, V. V.; Fedyanin, A. A.; Goodilin, E. A.; Ellenbogen, T.; Tenne, R.; Oron, D., Strong light–matter interaction in tungsten disulfide nanotubes. *Phys. Chem. Chem. Phys.* **2018,** 20, (32), 20812-20820.
7. Sinha, S. S.; Zak, A.; Rosentsveig, R.; Pinkas, I.; Tenne, R.; Yadgarov, L., Size-Dependent Control of Exciton–Polariton Interactions in WS_2 Nanotubes. *Small* **2020,** 16, (4), 1904390.
8. Washizu, E.; Yamamoto, A.; Abe, Y.; Kawamura, M.; Sasaki, K., Optical and electrochromic properties of RF reactively sputtered WO_3 films. *Solid State Ionics* **2003,** 165, (1-4), 175-180.
9. Višić, B.; Yadgarov, L.; Pogna, E. A. A.; Dal Conte, S.; Vega-Mayoral, V.; Vella, D.; Tenne, R.; Cerullo, G.; Gadermaier, C., Ultrafast nonequilibrium dynamics of strongly coupled resonances in the intrinsic cavity of WS_2 nanotubes. *Phys. Rev. Res.* **2019,** 1, (3), 033046.
10. S.S. Sinha, B. V., A. Byregowda and L. Yadgarov, Dynamical nature of exciton-polariton coupling in WS_2 nanoparticles. *Isr. J. Chem.* **2022,** 62, e202100128.
